# Feed restriction affects follicular development by regulating cell cycle progression in porcine mural granulosa cells

**DOI:** 10.1186/s40104-026-01464-1

**Published:** 2026-07-21

**Authors:** Qi Yu, Anna F. Bekebrede, Natasja N. G. Costermans, Nicoline M. Soede, Katja J. Teerds, Jaap Keijer

**Affiliations:** 1 Human and Animal Physiology, Wageningen University, De Elst 1, Wageningen, 6708WD The Netherlands; 2 Adaptation Physiology Group, Wageningen University, De Elst 1, Wageningen, 6708WD The Netherlands

**Keywords:** Cell cycle, Feed restriction, Follicular development, IGFBP system, Mural granulosa cells

## Abstract

**Background:**

Mammalian reproductive outcomes are influenced by whole-body energy status, especially in high-prolific sows. During lactation, high energy demands often lead to a severe negative energy balance (NEB), which compromises subsequent reproductive performance. The molecular processes underlying the sustained impact of lactational energy insufficiency on post-weaning follicular development are not well understood. We hypothesize that this energy shortage disrupts the development of mural granulosa cells (GCs), ultimately compromising oocyte developmental competence. We aimed to delineate the molecular processes by which energy status regulates mural GC development employing a nutritional intervention.

**Results:**

Over the 14 days of feed restriction, sows receiving a restricted diet (RES; 3.25 kg/d) lost double the amount of body weight and loin muscle depth compared to full-fed sows (FF; diet 6.5 kg/d). Both ovarian weight and average size of the largest healthy follicle were significantly reduced. RNA-seq analysis identified 2,282 differentially expressed genes in RES GCs, of which 1,531 were upregulated and 751 were downregulated. The most enriched pathway was the cell cycle, corroborated by CDK1 immunostaining. RES GCs showed upregulation of key positive cell cycle regulators (e.g., *CCNA1/2*, *CCNB1–3*, *CCNE2*), while cell cycle inhibitor *CDKN1A* (P21) was downregulated. In contrast to FF GCs, in RES GCs several members of the IGFBP family, especially *IGFBP7*, strongly correlated with most cell cycle-related genes including *FOXO1*, *CDKN1A*, and follicle size exclusively in RES GCs, but not in FF sows. Proliferation markers (*PCNA* and *MKI67*) were also upregulated, while differentiation marker *CYP19A1* was downregulated.

**Conclusion:**

Our data suggest that a severe lactational NEB has a lasting impact on mural GCs after weaning, delaying their transition from proliferation to the terminal differentiation. We propose a potential regulatory axis in which IGFBPs, especially *IGFBP7*, may link energy metabolism and follicular development through the AKT-FOXO1-CDKN1A-cell cycle regulatory axis. These molecular alterations likely contribute to the compromised oocyte competence observed following energy restriction. Furthermore, our findings identified IGFBP7 as a candidate biomarker linking metabolic status to follicular development.

**Supplementary Information:**

The online version contains supplementary material available at 10.1186/s40104-026-01464-1.

## Introduction

Energy status and reproductive function are tightly interconnected in mammals [1]. This connection is clearly evident in litter-bearing species such as modern hyper-prolific sows, where continuous selection for large litter size has greatly increased their energy demands during lactation [2]. Primiparous sows have limited body reserves and a low feed intake capacity [3], making them more susceptible to a severe negative energy balance (NEB) during lactation. This often results in lower pregnancy rates and reduced litter sizes in subsequent parities, a phenomenon widely recognized as second litter syndrome [4]. Our previous study suggested that this compromised fertility is likely attributed to the impact of lactational energy insufficiency on follicular development post-weaning [5].

Follicular development is the foundation for oocyte quality and subsequent embryonic development [6]. After weaning, follicles undergo profound morphological remodeling, characterized by rapid expansion and follicular fluid accumulation [7]. Mural granulosa cells (GCs) synthesize steroid hormones, growth factors, energy substrates, and metabolites [8, 9]. Any disruption in energy availability may impair mural GC function, ultimately compromising oocyte maturation and the resumption of meiosis [10]. Although the correlation between energy insufficiency and reduced follicle size is documented [11], the underlying molecular mechanisms and the relevant changes in follicle development still remain unclear. Because ovarian follicles are composed of several cell types, precise molecular profiling of mural GCs is challenging. This complexity poses a significant barrier to achieving a high-resolution mechanistic understanding of how follicles might be arrested by lactation-induced energy insufficiency. Consequently, this knowledge gap hampers the development of strategies to prevent adverse outcomes. The aim of this study is therefore, to elucidate molecular pathways that operate in mural GCs, the gatekeepers of oocyte maturation and quality, and which are associated with follicular development. We hypothesize that upon release from lactational energy restriction, mural GCs undergo compensatory molecular responses ("catch-up processes"). By analyzing these responses, we aim to identify the specific biological processes suppressed by energy restriction and underscore their functional relevance in follicular development.

Traditional bulk RNA-seq averages signals across mixed cell types, while single cell RNA-seq (scRNA) offers single-cell resolution, but lacks spatial context. The well-defined boundary of mural GCs from surrounding cell types allows precise isolation via laser capture microdissection (LCM) [12]. By combining LCM with UMI-based RNA-seq, a method that minimizes technical noise and improves detection of low-abundance transcripts, allows us to specifically and comprehensively map energy restriction related molecular alterations in mural GCs. This insight into the biology of mural GCs’ responses to energy stress is essential for a comprehensive understanding of reproductive physiology. In addition, this research provides a theoretical basis for optimizing lactational diets and nutritional care within swine industry.

To induce a severe NEB, a 50% feed restriction (RES) is applied to sows during the last two weeks of lactation (d 10 to 24 (end)), while the control group is full-fed (FF). This period corresponds to peak milk production in modern sows, when energy demands are highest [13]. This restriction level was chosen as it effectively drives a robust catabolic state [14] and as similar reductions in voluntary feed intake are commonly observed under heat stress conditions in commercial swine production [15]. We investigated mural GCs on d 2 post-weaning as by this time a cohort of selected follicles undergoes rapid growth (transitioning from medium to large antral follicles) and actively synthesizes estradiol, prior to the preovulatory LH surge, and subsequent ovulation at approximately d 5–6 after weaning [16]. This time point captures the physiological and endocrinological characteristics of the mid-follicular phase [17] and thus provides an optimal window to evaluate the physiological and molecular characteristics associated with the "last effects" of a lactational NEB. For this we combined LCM with low-input RNA-seq obtain deep reads from limited amounts of high-quality RNA, followed by advanced data analysis and confirmatory immunohistochemistry experiments.

The following structured stepwise research approach was followed: To obtain an overview of changes, we employed GC specific RNA-seq and used all expressed genes as the basis for our analysis. After identifying the main affected process, we delineated the most relevant upstream pathway as well as upstream regulators of this pathway by correlation analyses. By correlation to follicle size, we substantiated the functional relevance of this pathway. Further substantiation came from confirmatory immunohistochemistry and the analysis of proliferation and differentiation markers. Finally, we provided a larger overview of signaling pathways linked to the core pathway for an easy access to the wider information of our unique study.

## Materials and methods 

### Animals

Thirty-six primiparous sows (TN70, a crossbreed of Large White and Norwegian Landrace (TopigsNorsvin, The Netherlands)) were housed at the Wageningen University animal facility CARUS from 2 weeks before parturition onwards. During this period until parturition, sows were fed twice daily (at 700 and 1600 h) and received 2.9 kg/d of a standard gestation diet (Standard sow feed, AgruniekRijnvallei, The Netherlands). Within 72 h after parturition, piglets were cross fostered to obtain similar litter sizes. During lactation, all sows were fed the same standard lactation diet containing approximately 9.3 MJ NE/kg, 156 g/kg CP, 8.9 g/kg lysine, and 7.69 g/kg SID lysine (Maxima lacto, AgruniekRijnvallei) divided over three meals a day (0700 h, 1300 h, and 1900 h). All sows received the same amount of diet until 2 weeks before weaning, starting at 2 kg/d at parturition and increasing with 0.5 kg/d from d 2 after parturition to reach 6 kg/d at d 10 of lactation, according to the TN70 feeding manual for primiparous sows. After d 10, 18 sows were fed a daily allowance of 6.5 kg (full-fed, FF), and 18 sows were fed 3.25 kg/d (feed-restricted, RES) for 2 weeks, until weaning (Fig. S1). The piglets were creep-fed. Sows had an average lactation length of 24.1 ± 0.3 d for both FF and RES. To mimic commercial practices and to assess whether lactational energy insufficiency has a negative influence on follicular development after weaning, all sows (FF and RES) were fed 3 kg/d from weaning until 48 h post-weaning, as described previously [5]. Day 2 after weaning is considered the mid-follicular phase, which was selected because it is a key stage to determine whether follicles will progress to maturity or undergo atresia [18]. Ovaries were collected and follicular size was measured of all visible antral follicles, as described before [10]. After this, the ovaries were immediately frozen in liquid nitrogen and stored at −80 °C until further analyses. Primarily for practical reasons, but also to enhance discriminatory power, 14 sows with the highest body weight loss from the RES group and 14 sows with the lowest body weight loss from the FF group were selected for this study (from the two original groups of 18 sows).

### Body weight, backfat depth, muscle depth

Body weight, back fat depth, and muscle depth were measured at different time points after arrival of the sows at the university animal facility CARUS, as described in detail by Costermans et al. [5].

### Mural granulosa cell collection

The right ovary of each sow was cut into two halves (Fig. S2). Mural GCs were collected from the largest healthy follicle using LCM after the following procedure. To ensure that the cryo-sections contained (almost all) large antral follicles visible on the surface of the ovary, frozen ovaries from each sow were cut into three planes [19]. For follicle size measurement, each cutting plane was photographed against a grid paper as a scale reference using a Nikon D3300 camera. Follicle size was then determined as the largest macroscopically visible diameter using ImageJ software (v1.52, National Institute of Health, USA). Subsequently, to determine if a follicle was healthy or atretic, immunohistochemistry was applied using an antibody against the apoptosis marker cleaved-caspase 3. Cryosections (7 µm) were fixed in 4% (v/v) paraformaldehyde for 10 min and washed in Milli-Q water. The sections were incubated in 0.75% (w/v) glycine in Tris-buffered saline (TBS) for 30 min and rinsed 3 × 5 min in TBS before a 1 h incubation in 5% (v/v) normal goat serum. The sections were incubated overnight at 4 °C with the primary polyclonal rabbit anti‐cleaved‐caspase 3 antibody (lot number 47, #9661S, Cell Signaling Technology, USA) diluted 1:400 (v/v) in TBS‐BSA‐c (Aurion, The Netherlands). Next, sections were rinsed with TBS 5 × 5 min, and incubated for 1 h at room temperature with a goat‐anti‐rabbit Alexa Fluor 488 secondary antibody (#A-11012, ThermoFisher, USA) diluted 1:200 (v/v) in TBS‐BSA-c. After 3 × 5 min washing with TBS and incubation with 4′,6-diamidino-2-phenylindole (DAPI) (#28718-90-3, Sigma-Aldrich, USA) for 10 min, slides were rinsed with TBS for 3 × 5 min and mounted, after which images were captured by microscopy (DM6B, Leica, Germany) (Fig. S3). Follicles in which more than 5% of the mural GCs stained positive for cleaved-caspase 3 were considered atretic and excluded from further analysis.

After identification of the largest healthy follicle of each animal, mural GCs were isolated as follows: 30 μm cryo-sections were cut by a cryostat microtome (CM3050 S, Leica, Germany) and mounted on polyethylene naftelato (PEN) membrane framed slides (#11600288, Leica, Germany). Subsequently, the sections were fixed in 100% ethanol for 3 × 1 min, followed by a 10 s counterstain with Mayer’s hematoxylin (#H9627-25G, Sigma-Aldrich, USA). Next, the slides were rinsed with RNase-free water 3 × 15 s, and 100% ethanol for 3 × 1 min before being air-dried for 2 min. Mural GCs (around 30,000 cells/sample) were captured under 20 × magnification using an LCM microscope (Leica LMD 7000, Germany) and transferred into 0.5 mL RNase-free Qubit assay tubes containing 200 µL of lysis buffer (#217684, Qiagen, Germany). After incubation at room temperature for 30 min, the tubes were placed on dry ice before being transferred to a −80 °C freezer.

### RNA isolation and cDNA library preparation

Total RNA of mural GCs was extracted using the TRIzol method (#15596018, ThermoFisher, USA) following the manufacturer’s instructions. The RNA yield and concentration were determined using the Nanodrop spectrophotometer (IsoGen Life Science, the Netherlands). Additionally, the RNA Integrity Number (RIN) was assessed using the Agilent 2100 Bioanalyzer (Agilent, USA). According to Kerman et al. [20], the RIN of LCM RNA samples is categorized as excellent for RIN ≥ 7, good for 5 ≤ RIN < 7, and poor for RIN < 5. In this study, the mean RIN was 7.30 ± 0.60 (± SD), and none of the RNA samples used in this study had a RIN below 5 (Table S1). One individual from the FF sow group was excluded prior to downstream analysis due to an abnormal RIN pattern. Next, rRNAs were removed from the total RNA, followed by fragmentation to 200–500 nucleotides. First-strand cDNA was synthesized using a random hexamer-primer, while dTTP was substituted by dUTP during the synthesis of the second strand. The short fragments were purified and treated with elution buffer to facilitate end repair, followed by the addition of a single nucleotide A (adenine). After that, the adapters were connected to the fragments, and UNG (uracil-N-glycosylase) was used to degrade the second strand. After agarose gel electrophoresis, the suitable fragments were selected for several rounds of PCR amplification.

### Sequencing and data processing

The final PCR products were sequenced (150 bp paired-end reads) using the DNBseq platform (BGI-Shenzhen, China) with an average depth of 38 million reads per sample. After sequencing, base calling (BCL) files were converted to FASTQ files using bcl2fastq2 (Illumina, USA). The “dirty” raw reads, defined as reads that contain adapters, high content of N bases, and low-quality reads, were removed using SOAPnuke by BGI, with the following parameter setting (-l 15 -q 0.2 -n 0.05 -i). After removal of low-quality reads, an average of 36.5 million and 38.4 million clean reads were obtained from the FF and RES libraries, respectively. Clean reads were checked for sequence performance with FastQC (v0.11.9) and MultiQC (v1.14). The average Q30 score was approximately 94%. The clean reads were aligned to the pig reference genome (scrofa.Sscrofa11.1) using HISAT2 (v2.2.1) [21], applying the following parameters: --sensitive --no-discordant --no-mixed -I 1 -X 1000. In total, 80% ± 1.6% of the reads could be uniquely mapped to the pig reference genome, and 4.6% (1,655,389) to 11.9% (4,710,010) of the reads matched multiple locations (Table S2), showing that qualitatively and quantitatively good sequences were obtained from the LCM-captured mural GCs. The uniquely mapped reads were extracted using Samtools (v1.16.1), followed by deduplication using UMI-tools (v1.1.4) [22] with default parameters. This step was performed to eliminate duplicates caused by PCR amplification before sequencing to obtain accurate quantification. Gene counts were obtained using FeatureCounts (v1.6.2) [23] with the parameters: -p -T4 -t exon -g gene_id -a -o. Genes with an average read count of less than 5 across all animals were discarded, and normalization was performed using the negative binomial distribution implemented in the Bioconductor package DESeq [24]. Principal components analysis (PCA) was plotted using the R package ggplot.

### Analysis of differential gene expression and functional enrichment

Once the mRNA counts were normalized, the DESeq2 R package was used to identify DEGs with the FF group as the control. Gene set enrichment analysis (GSEA) was performed using gene ranking based on a composite score derived from the log_2_ fold change (log_2_FC) and adjusted *P*-value (*P*_adj_) [25] to identify enriched Gene Ontology (GO) functions and Kyoto Encyclopedia of Genes and Genomes (KEGG) pathways according to previously described protocols [26]. The normalized enrichment score (NES) of each gene set was estimated by the amount of over-representation of members of the gene set towards the top or bottom of the ranked gene list by applying weighted Kolmogorov–Smirnov statistics [27]. Results with a *P*_adj_ < 0.05 and |NES| > 1 of GSEA were considered significantly enriched and visualized as a bubble plot using the GseaVis package [28] in R software.

### Gene co-expression network construction

The co-expression network of normalized gene expression counts (> 5 counts) was constructed using the weighted correlation network analysis (WGCNA) [29] (v1.73) in R. Briefly, the normalized data were subsequently log transformed as suggested in the WGCNA manual to build a signed network. Pairwise Pearson’s correlations among all genes were calculated to create an adjacency matrix. A soft threshold power was set at β = 9, corresponding to a scale-free topology index (R^2^) of 0.8. The adjacency matrix was employed to compute the Topological Overlap Measure (TOM), based on which modules of co-expressed genes were identified using the dynamic tree cut algorithm. Each module was arbitrarily assigned a unique color label. The modules were constructed with a cut height of 0.25 and a minimum module size of 14,000 genes.

### Expression and cellular localization of CDK1 and CDKN1A

To validate the pathway analysis results, immunohistochemistry was performed using CDK1, a specific marker for cell cycle progression. The largest healthy follicles from six ovaries in each group were cryosectioned and mounted on Superfrost Plus glass slides (MenzelGläser, Germany). The procedure followed the above-described method for the detection of cleaved-caspase 3 with minor modifications. In this case, the primary antibody was replaced by either an anti-CDK1 antibody (lot number 1023303–7, #ab32094, Abcam, UK), diluted 1:500 (v/v) in TBS‐BSA-c, or an anti-CDKN1A (anti-p21 antibody, lot number 1002684–26, #ab109199, Abcam, UK) diluted 1:200 (v/v) in TBS‐BSA-c. Isotype IgG was used as a negative control. The areas of DAPI-stained nuclei were segmented using the StarDist model in ImageJ software (v1.52). The mean staining intensity, calculated as the sum of gray values in all pixels divided by the number of selected pixels, was then quantified separately for the nuclear and cytoplasmic regions of mural GCs.

### Statical analysis

Statistical analysis was performed using GraphPad Prism (v9.31). Data were tested for normality and results were presented as mean ± standard deviation (SD). A Student’s *t*-test was used for comparisons, with *P* < 0.05 considered statistically significant and *P* < 0.10 considered indicative of a trend. To identify potential driver genes and their downstream signaling pathways that may regulate cell cycle progression, Pearson correlation (for normally distributed data) and Spearman rank correlation (for non-normally distributed data) were performed between DEmRNA candidates and follicle size. No covariates were used in this study. The plots were generated using GraphPad Prism.

## Results

### Feed restriction reduces body weight, muscle mass, and follicular development

**Fig. 1:**
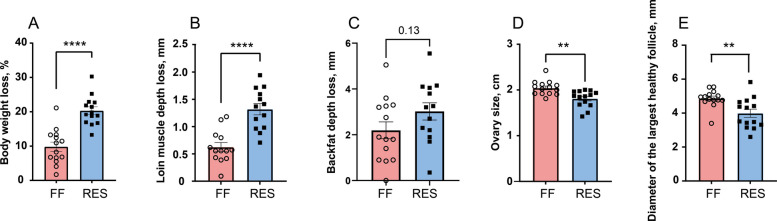
Phenotypic characteristics of full-fed or feed-restricted sows. Comparison of body weight loss (**A**), loin muscle (**B**), backfat (**C**) ovary size (**D**) and follicle size of the largest healthy follicles (**E**) between the FF and the RES sows. ^**^P < 0.01, ^****^P < 0.0001

Before the dietary intervention, on d 10 of lactation, there were no significant differences between the FF and RES sows in body weight, loin muscle depth, or backfat depth (Table 1). After the two-week period of lactational feed restriction, RES sows had lost almost the double amount of body weight (%) compared to FF sows (20.3% ± 5.1% vs. 9.8% ± 4.3%, *P* < 0.0001) (Fig. 1A). The loin muscle depth loss of RES sows was significantly higher during lactation compared with the FF sows (1.3 ± 0.3 mm vs. 0.6 ± 0.4 mm, *P* < 0.0001) (Fig. 1B), whereas no significant difference was observed in backfat loss between the two groups (3.0 ± 1.4 mm vs. 2.2 ± 1.4 mm, *P* = 0.13) (Fig. 1C). The ovary size of RES sows was smaller compared to FF sows (1.81 ± 0.18 cm vs. 2.04 ± 0.15 cm, *P* = 0.0012) (Fig. 1D), and not surprisingly the diameter of the largest healthy follicle out of the pool of 15 largest follicles per ovary was significantly smaller in RES sows (4.0 ± 0.9 vs. 4.9 ± 0.5 mm for FF sows, *P* = 0.0042) (Fig. 1E).

**Table 1:** Sows’ metabolic and follicle characteristics before and after two weeks of feed restriction

Sow metabolic state	FF^a^	RES^b^	*P*-value
Body weight on d 10 of lactation, kg	201.0 ± 18.2	192.1 ± 17.5	0.20
Body weight at weaning, kg	174.3 ± 19.6	161.4 ± 15.0	0.0003^***^
Loin muscle depth on d 10 of lactation, mm	5.2 ± 0.6	5.2 ± 0.6	0.99
Loin muscle depth at weaning, mm	4.6 ± 0.6	3.9 ± 0.5	0.0018^**^
Backfat depth on d 10 of lactation, mm	12.0 ± 1.7	12.0 ± 2.6	0.97
Backfat depth at weaning, mm	9.7 ± 1.5	8.7 ± 2.6	0.24
Sow follicle parameters			
Ovary size, cm	2.04 ± 0.15	1.81 ± 0.18	0.0012^**^
Diameter of the largest healthy follicles, mm	4.9 ± 0.5	4.0 ± 0.9	0.0042^**^

^a^Full-fed group

^b^Restricted-fed group

***P-value < 0.001; **P-value < 0.01. The data represent 14 RES sows with the highest body weight loss and 14 FF sows with the lowest body weight from a previously published study, that did not investigate GCs or molecular processes, encompassing two times 18 sows [5]

### Marker gene-based purity validation and distinct mRNA expression patterns between FF and RES groups

**Fig. 2:**
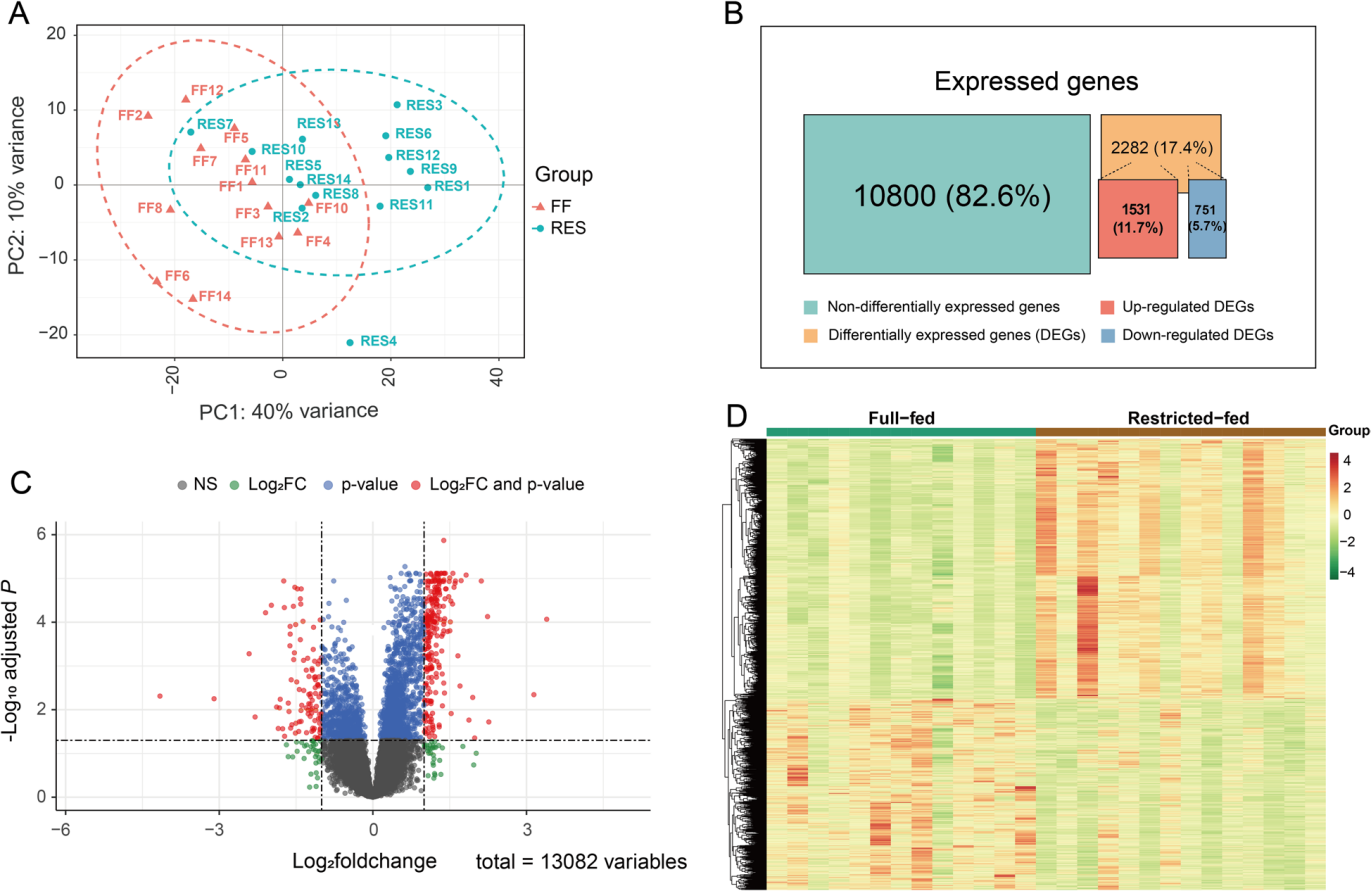
Overview of transcriptome profiling in mural granulosa cells from full-fed and restricted sows. **A** Principal component analysis (PCA) of GCs transcriptome gene counts. The red triangles represent the FF sows, and the green dots represent the RES sows. PC1 and PC2 are principal component 1 and 2, respectively. **B** Statistical analysis of GCs expressed genes. Non-differentially expressed genes in blue field. All DEGs (*P*_adj_ < 0.05) in orange field, with up-regulated DEGs in red field and down-regulated DEGs in blue field. The FF group was used as the reference group. **C** The volcano plot of all GCs protein coding genes comparing the FF and RES groups. The *x*-axis represents the log_2_fold change, and the *y*-axis displays −log_10_ (adjusted *P*-value). The cutoff is log_2_|FC| > 1 and adjusted *P*-value < 0.05. Each dot represents one gene. **D** Heatmap of the 2,282 GCs DEGs between the FF and the RES group

High-quality RNA-seq data were obtained from LCM-captured mural GCs. Next, a total of 13,082 protein-coding genes were detected in both groups, and their normalized counts were used for GC purity assessment, PCA, and differential expression analysis. To assess the purity of the isolated mural GCs, we analyzed the expression levels of markers for different follicular cell types. Although certain theca cell markers (*CYP11A1*, *CYP17A1*, *RET*, *COL14A1*, *PTCH2*, *DHCR24*, and *BGN*) [30, 31] were detectable, their expression levels were substantially lower than those of GC markers (*CYB5A*, *INHA*, *INHBA*, *INHBB*, *GSTA1*, *FST*, and *VCAN*) [30, 32]. Markers for immune cells (*CD68*, *CD16,* and *CD19*) [33], blood vessels (*PDGFRB* and *CDH5*) [33], and lymphatic vessel cells (*LYVE1*) [33] were barely detectable (Fig. S4). While the PCA plot showed that the two groups did not separate completely, PC1 explained 40%, and PC2 explained 10% of the total variance in the dataset (Fig. 2A). Furthermore, DEG analysis revealed 2,282 (17.4% of all expressed genes) as significantly differentially expressed between the two groups (*P*_adj_ < 0.05). Among these DEGs, 1,531 mRNAs (67.1%) were up-regulated, and 751 mRNAs (33.9%) were down-regulated in RES sows compared to FF sows (Fig. 2B). The volcano plot showed that 246 upregulated DEGs had a fold-change larger than 2 (FC > 2), of which six displayed a FC > 4, while 101 downregulated DEGs had a FC > 2, of which five had a FC > 4 (Fig. 2C). A heatmap of 2,282 DEGs was generated, showing a difference between the FF and RES groups, providing insight in variation among the animals (Fig. 2D).

### Integrated functional enrichment and weighted correlation network analysis

**Fig. 3:**
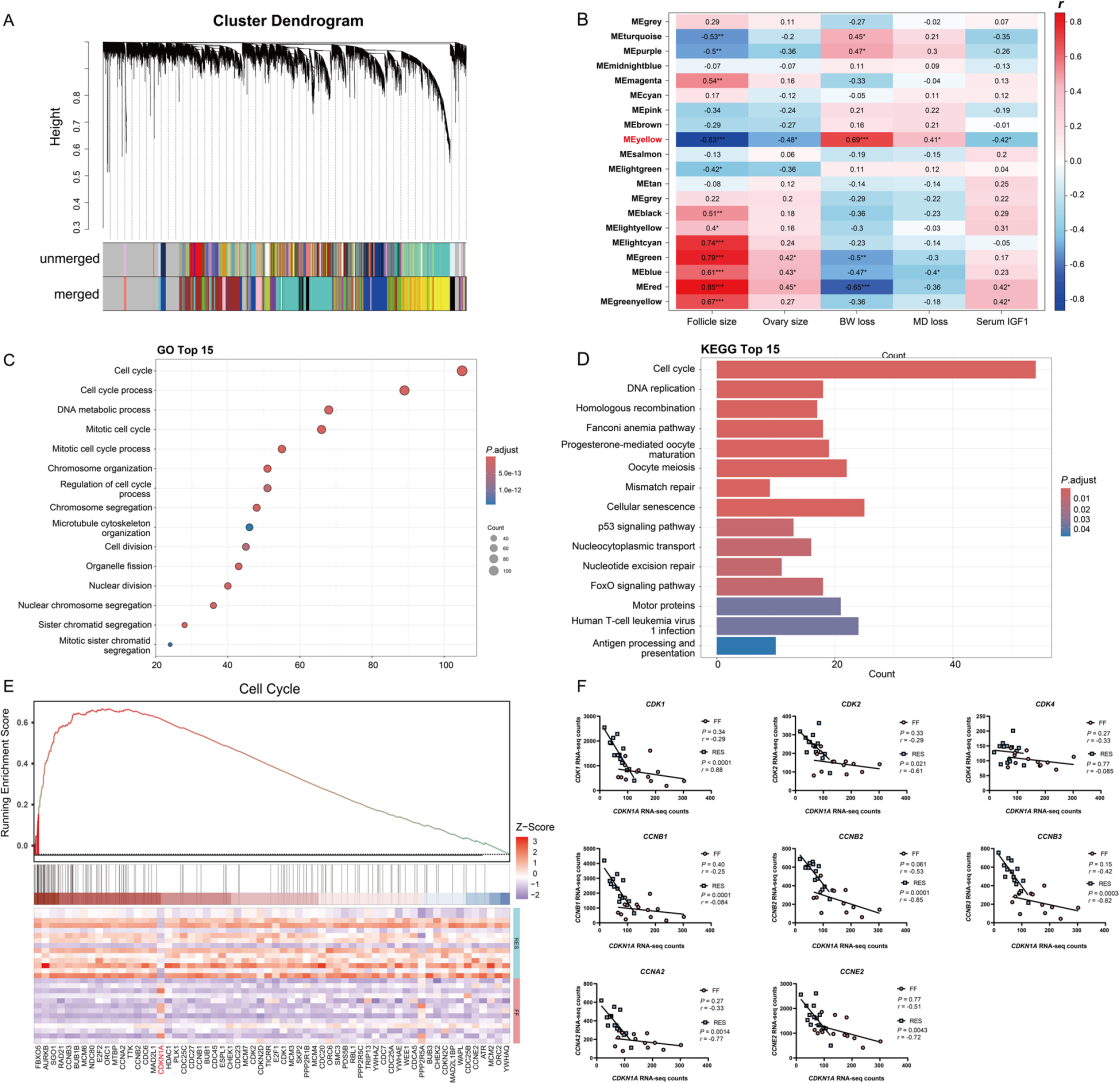
Functional analysis of gene expression and immunohistochemistry of full-fed and feed-restricted sow GCs. **A** Weighted gene correlation network analysis of the 27 RNA-seq sample datasets. The genes corresponding to branches of the same color are classified as the same gene module. **B** Gene modules associated with follicle size, ovary size, body weight loss, muscle depth loss and serum IGF1 levels. Numbers represent corresponding correlation coefficient; colors represent positive and negative correlation. **C** The top 15 enriched GO terms for genes in the module MEYellow. **D** The top 15 enriched KEGG pathways for genes in the module MEYellow. **E** The GESA enrichment plot of the cell cycle pathway. Each column represents one gene. Each row represents one individual. **F** Relations between *CDKN1A* mRNA expression and positive cell cycle regulators. Each filled dot represents an individual. The *r* represents the Pearson correlation coefficient

The gene expression profile was analyzed using different data annotations (GO and KEGG) and approaches (WGCNA and GSEA). We employed WGCNA on RNA-seq data of GCs and identified 20 gene modules (Fig. 3A). Notably, the MEYellow module was the only module significantly associated with all phenotype outcomes (Fig. 3B). GO and KEGG enrichment analyses revealed that genes in the MEYellow module were predominantly involved in cell cycle-related processes (Fig. 3C and D). To underpin the above results, we additionally employed a GSEA approach. Many of the significantly enriched GO terms with high gene counts were strongly and positively associated with cell cycle processes in RES GCs, such as “cell cycle” and “cell cycle process”. Additionally, one GO term, "DNA metabolic process", was positively linked to metabolism. Conversely, “tissue development” and “animal organ morphogenesis” were downregulated in RES GCs (Fig. S5). Similarly, using KEGG GSEA, “cell cycle” was most positively enriched in RES GCs (NES = 2.6), followed by “DNA replication” (NES = 2.3). Follicular development-related pathways, such as “oocyte meiosis” and “progesterone-mediated oocyte maturation”, were also enriched. (Fig. S6), supporting the WGCNA enrichment results. The cell cycle pathway exhibited a positive running enrichment score, and genes included in this pathway revealed a consistent, significantly higher expression of most key cell cycle genes in RES GCs (Fig. 3E). This included the cellular communication network factor (CCN) family (*CCNB1–3*, *CCNA2*, *CCNE2*), the CDC-like kinase (CDC) family (*CDCA5*, *CDC6*, *CDC7*, *CDC20*, *CDC23*, *CDC27*, *CDC25B-C*, *CDC45*), and the cyclin-dependent kinase (CDK) family (*CDK1*, *CDK2*, *CDKN2C*). Notably, one cyclin-dependent kinase inhibitor (CDKN), *CDKN1A*, showed an inverse pattern, being significantly downregulated in RES GCs (*P* < 0.0001). Correlation analysis revealed that *CDKN1A* was negatively correlated with members of the CDK and CCN families exclusively in the RES group (Fig. 3F).

### Validation of key cell cycle regulators CDK1 and CDKN1A

**Fig. 4:**
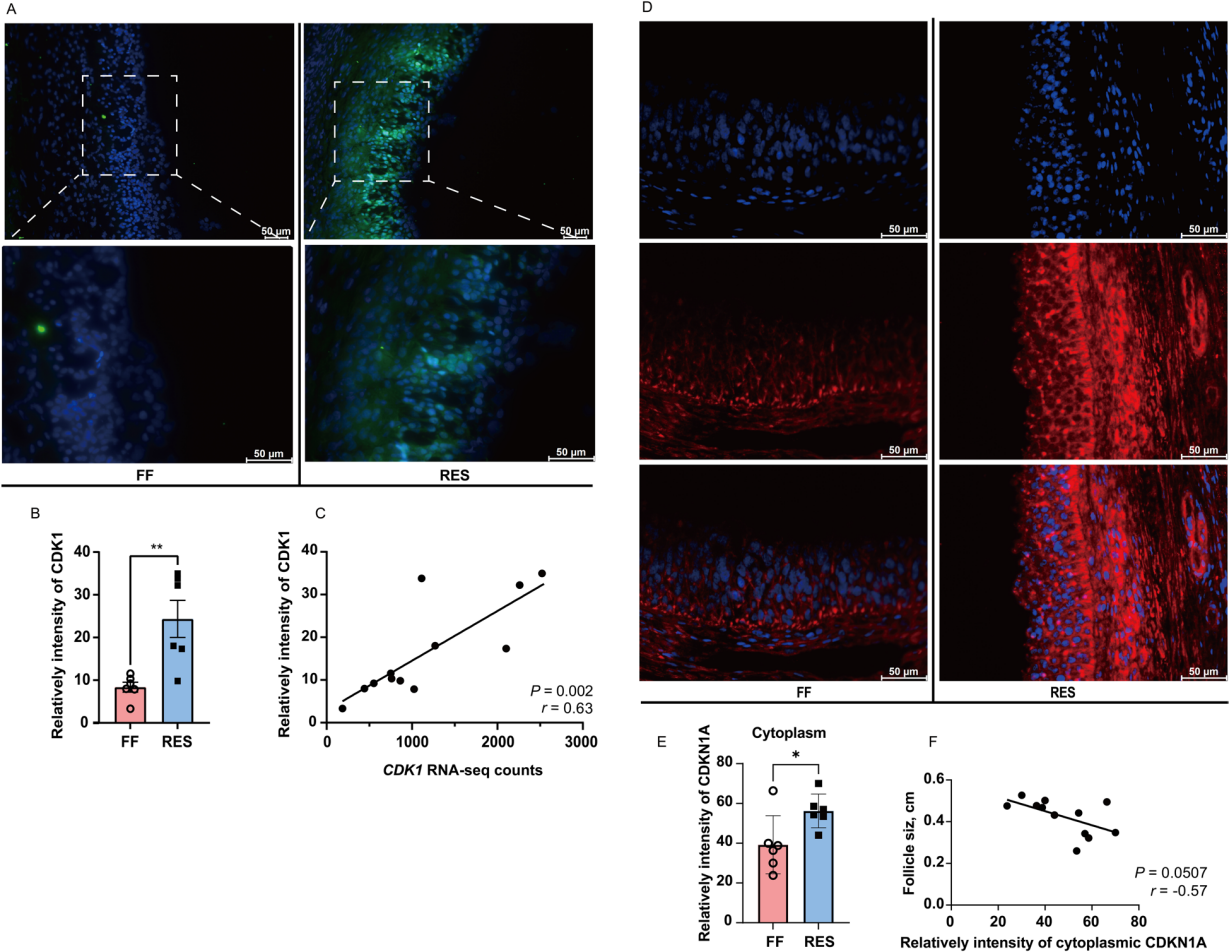
Validation of key cell cycle regulators CDK1 and CDKN1A. **A** Representative images of immunohistochemistry staining for the cell cycle marker CDK1 in mural GCs of the target follicles. The FF GCs (upper panel) and the RES GCs (lower panel) are at 20 × (left) and 40 × (right) magnification. **B** Quantitative analysis of CDK1 immunohistochemistry staining in FF and RES follicles (*n* = 6; randomly selected). ^**^P < 0.01. **C** Correlation between *CDK1* mRNA expression and mean immunohistochemistry staining intensity. The *r* represents Pearson correlation coefficient. **D** Representative images of immunohistochemistry staining for CDKN1A (left: FF; right: RES, 20 × magnification). **E** Quantitative analysis of immunohistochemistry staining for CDKN1A. ^*^P < 0.05. **F** Correlation between *CDKN1A* mRNA expression and mean immunohistochemistry staining intensity. The *r* represents Pearson correlation coefficient

To validate the above enrichment results, CDK1, a common marker for cell cycle progression, was quantified by immunohistochemistry (Fig. 4A). The protein level of CDK1 in mural GCs was significantly higher in RES sows compared to FF sows (*P* = 0.0052) (Fig. 4B). Furthermore, Pearson correlation analysis showed a strong, positive correlation between the mRNA and protein levels of CDK1, confirming the RNA-seq data that cell cycle progression was upregulated in RES GCs (Fig. 4C). Immunofluorescent staining of CDKN1A showed that it was localized in the cytoplasm (Fig. 4D). Since CDKN1A has different roles depending on it being localized in the nucleus or in the cytoplasm, we separately quantified its levels in the cytoplasm and the nucleus. CDKN1A was not detected in GC nuclei. In the cytoplasm, where its function is largely opposite of the nuclear cell cycle inhibitory function, CDKNA1 levels were significantly higher in RES GCs (*P* = 0.033) (Fig. 4E). Furthermore, cytoplasmic CDKNA1 levels tended to negatively correlate with follicle size (Fig. 4F). The higher cytoplasmic levels in RES GCs are functionally in agreement with the observed decreased gene expression. Together, the above results revealed that the lactational whole-body energy status caused a significant difference in the cell cycle process between RES and FF GCs, likely driven by CDKN1A.

### Potential upstream pathways and regulators in modulation of CDKN1A

**Fig. 5:**
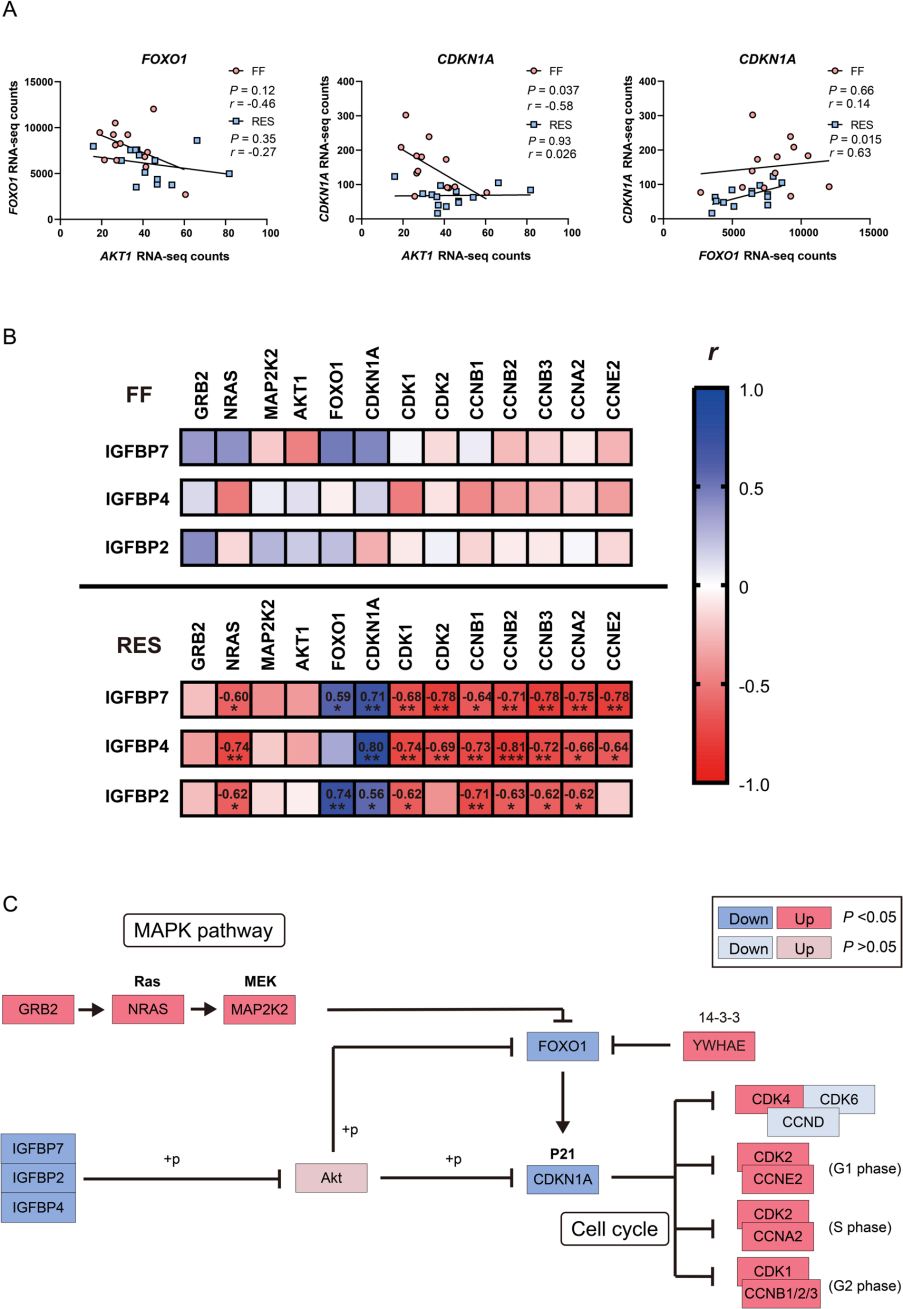
Gene expression of the AKT-FOXO1-CDKN1A-cell cycle regulatory pathway in GCs from full-fed and feed-restricted sows. **A** Correlations among *FOXO1*, *AKT1*, and *CDKN1A*. Each filled dot represents an individual (*n* = 27). The *r* represents Pearson correlation coefficient. **B** Correlation matrix of IGFBPs and key genes in the regulatory axis. Red indicates negative correlations, blue to positive correlations. **C** Scheme of the proposed GC regulatory mechanism. Significantly upregulated genes (*P*_adj_ < 0.05) are shown in red. Light red indicates upregulated genes that are not statistically significant. Significantly downregulated genes (*P*_adj_ < 0.05) are shown in blue. Light blue indicates downregulated genes that are not statistically significant. The FF group was used as the reference group

It is well established that AKT is a negative regulator of CDKN1A [34]. In addition, FOXO1 has been reported to be able to regulate CDKN1A by binding to its promoter [35]. The DEG data showed that *FOXO1* was significantly downregulated (*P* = 0.029), while *AKT1* showed a trend toward upregulation in RES GCs (*P* = 0.054). Therefore, we examined to what extent the central regulators *AKT1*, *FOXO1*, and *CDKN1A* were correlated. *FOXO1* was positively correlated with *CDKN1A* only in the RES group, whereas *AKT1* was not correlated with either *CDKN1A* or *FOXO1* at the gene expression level (Fig. 5A). We then focused on upstream regulators of AKT and FOXO1, as our previous research suggested that the IGF signaling pathway plays a vital role in linking metabolism and follicular development [5]. Broad correlation analyses among members of the IGF system, *AKT1*, *FOXO1*, *CDKN1A*, and cell cycle regulators were performed. Interestingly, several IGFBP family members exhibited significant and strong correlations with *FOXO1*, *CDKN1A*, and key cell cycle regulators, specifically in the RES group (Fig. 5B). Notably, *IGFBP4* and *IGFBP7* showed significant correlations with *CDK2* and *CCNE2* within the RES group, while only *IGFBP7* was correlated with *FOXO1*. These results indicated that these IGFBPs may act as potential upstream regulators. Accordingly, we proposed that the IGFBPs-FOXO-CDKN1A-cell cycle is a regulatory axis triggered by feed restriction to modulate cell cycle progression in GCs (Fig. 5C). As the only member of the IGFBP family, IGFBP7 was also presented in the WGCNA MEYellow module, enforcing that it likely activates the downstream pathway and ultimately regulates cell cycle progression. Besides, the MAPK pathway may participate in the regulation of *FOXO1*, because several members like *NRAS*, dual specificity mitogen-activated protein kinase kinase 2 (*MAP2K2*), and ribosomal protein S6 kinase (*RPS6KA1*) were differentially expressed, and both *NRAS* and *MAP2K2* showed significant correlations with *FOXO1* (*P* = 0.015; *P* = 0.032, respectively; data not shown) in RES GCs.

### Correlations between follicle size and IGFBPs, FOXO1, CDKN1A, and cell cycle regulatory axis

**Fig. 6:**
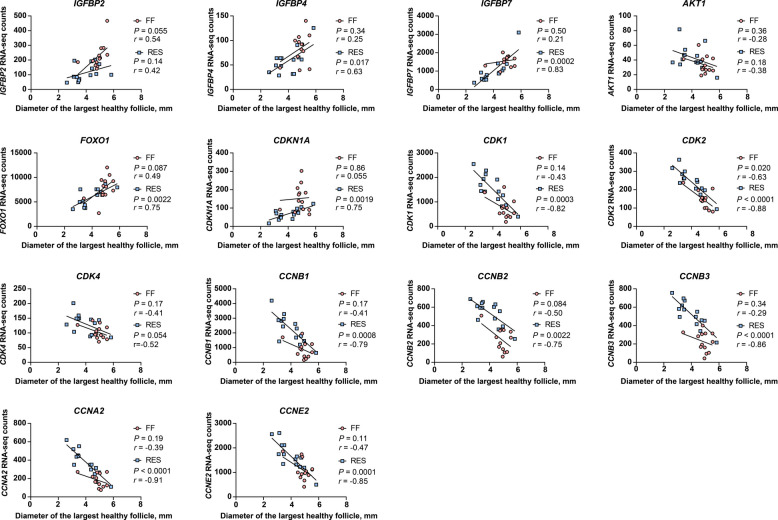
Association of gene expression of IGFBPs-AKT-FOXO1-CDKN1A-cell cycle regulators with follicle size. Correlations between the follicle size of the target follicles and *IGFBP7*, *IGFBP2*, *IGFBP4*, *AKT1*, *FOXO1*, *CDKN1A*, *CDK1*, *CDK2*, *CDK4*, *CCNB1*, *CCNB2*, *CCNB3*, *CCNA2*, *CCNE2*. Each filled dot represents an individual (*n* = 27). The *r* represents Pearson correlation coefficient

To confirm our proposed regulatory axis, we performed correlation analysis between follicle size and core members of this axis. These analyses revealed strong positive correlations between follicle size and *IGFBP*4 (*r* = 0.63), *IGFBP7* (*r* = 0.83), *FOXO1* (*r* = 0.75) and *CDKN1A* (*r* = 0.75). In contrast, strong negative correlations were observed between follicle size and *CDK1* (*r* = −0.82), *CDK4* (*r* = −0.52), *CCNB1* (*r* = −0.79), *CCNB2* (*r* = −0.75), *CCNB3* (*r* = −0.86), *CCNA2* (*r* = −0.91) and *CCNE2* (*r* = −0.85) (Fig. 6) in RES GCs, but not in FF GCs. These results indicate that the regulatory axis activated by energy restriction promotes GC proliferation, while the GCs in the larger, further developed follicles in FF sows are less proliferative (and more differentiated). To substantiate this, we analyzed proliferation and differentiation markers. Proliferation markers proliferating cell nuclear antigen (*PCNA*) and Ki-67 (*MKI67*) showed significantly higher gene expression levels in RES GCs, whereas the gene expression of P450 aromatase (*CYP19A1*), a differentiation marker, was significantly lower in RES GCs compared to FF GCs. Inhibin B (*INHBB*) expression was significantly higher in RES GCs. *INHBB* typically peaks in smaller antral follicles when the follicles in this study were harvested. In agreement, angiopoietin 1 (*ANGPT1*) expression, which is usually higher in larger antral follicles [19], was significantly lower in RES GCs (Fig. S7). Together, these findings imply that RES GCs were more proliferative and exhibited delayed differentiation compared to FF GCs.

### Both glycolytic and mitochondrial metabolism are upregulated to support GC growth in RES sows

**Fig. 7:**
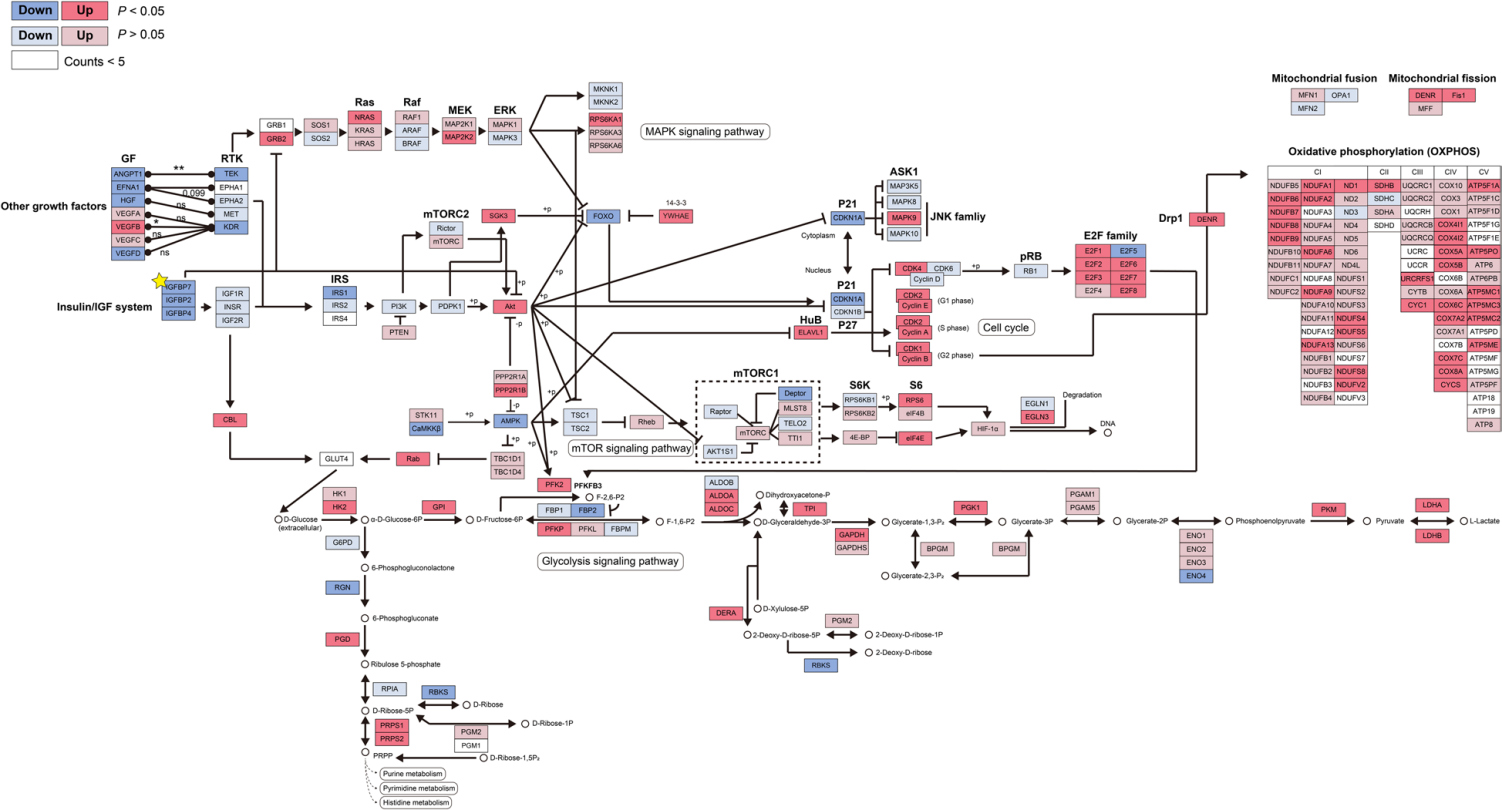
A comprehensive overview of the gene-expression based changes in FF and RES GCs. Significantly upregulated genes (*P*_adj_ < 0.05) are shown in red. Light red indicates upregulated genes that are not statistically significant. Significantly downregulated genes (*P*_adj_ < 0.05) are shown in blue. Light blue indicates downregulated genes that are not statistically significant. The FF group was used as the reference group

Proliferation is generally associated with increased glycolysis [36]. Analysis of DEGs indeed revealed an upregulation of glycolysis in RES GCs compared to FF GCs. Several genes encoding rate-limiting glycolytic enzymes were significantly upregulated, including 6-phosphofructo-2-kinase/fructose-2,6-bisphosphatase-3 (PFKFB3), hexokinase-2 (HK2), pyruvate kinase (PKM), and lactate dehydrogenase (LDH) (Table 2). A more extensive analysis displayed a full upregulation of the glycolysis pathway (Fig. 7 provides a comprehensive overview). While a shift from oxidative to glycolytic metabolism is considered a feature of proliferative (cancer) cells [36], oxidative phosphorylation (OXPHOS) also appeared to be elevated in RES GCs, with approximately 62% (105) of core OXPHOS-related genes (169 in total) being significantly upregulated (Fig. 7). Additionally, 54% of Mitocarta 3.0 mitochondrial genes unrelated to OXPHOS were significantly upregulated (Table S3), showing that mitochondrial metabolism as a whole was upregulated. For example, the ribosome, citrate cycle (TCA cycle), and metabolically related pathways were upregulated (Fig. S8). This suggests that activation of glycolytic and mitochondrial metabolism is not mutually exclusive, and stimulation of both might provide more energy and building blocks to meet the high demands of RES GCs to catch up growth [37].

**Table 2:** Expression levels of pure mural granulosa cell glycolysis pathway genes

Gene ID	Gene symbol	Log_2_FoldChange	Expression (counts)	*P*-value
ENSSSCG00000010253	HK1	0.095	186.4	0.48
ENSSSCG00000008261	HK2	1.1	20.7	0.019
ENSSSCG00000002873	GPI	0.22	731.5	0.11
ENSSSCG00000031174	FBP1	0.12	13.0	0.60
ENSSSCG00000010947	FBP2	−1.1	33.6	0.00015
ENSSSCG00000011133	PFKFB3	0.87	83.4	0.0022
ENSSSCG00000011158	PFKP	0.87	9.8	0.015
ENSSSCG00000028197	PFKL	0.19	22.0	0.40
	FBPM		< 5	
ENSSSCG00000032556	ALDOA	0.53	1,041.7	0.0069
ENSSSCG00000038300	ALDOB	−0.12	21.7	0.51
ENSSSCG00000017759	ALDOC	0.81	41.2	0.0016
ENSSSCG00000000685	TPI1	0.47	367.5	0.016
ENSSSCG00000000694	GAPDH	0.43	5,105.0	0.018
ENSSSCG00000002888	GAPDHS	0.27	11.6	0.29
ENSSSCG00000039425	BPGM	0.026	413.6	0.89
ENSSSCG00000012440	PGK1	0.53	1,570.7	0.0021
	PGAM1		< 5	
ENSSSCG00000035618	PGAM5	0.18	154.6	0.099
ENSSSCG00000022343	ENO1	0.40	1705.2	0.21
ENSSSCG00000028373	ENO2	0.018	11.9	0.94
ENSSSCG00000017904	ENO3	0.075	38.0	0.73
ENSSSCG00000010664	ENO4	−0.57	27.4	0.00097
ENSSSCG00000001930	PKM	0.55	1642.5	0.0024
ENSSSCG00000013366	LDHA	0.93	1,311.4	0.0020
ENSSSCG00000032557	LDHB	0.37	64.5	0.018

**Fig. 8:**
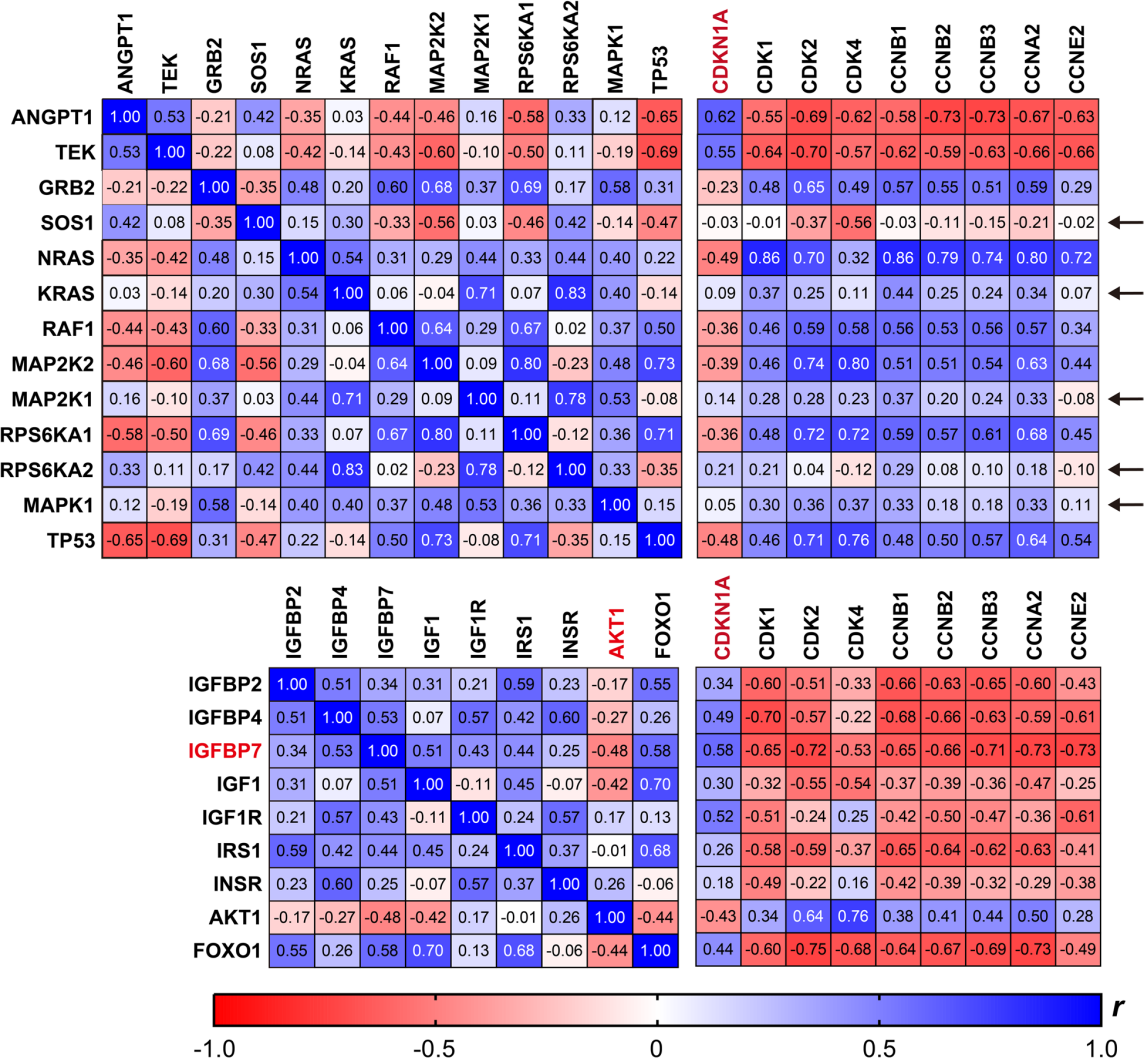
Correlation matrix of GC gene expression of MAPK, PI3K-AKT and cell cycle regulators. Pearson's correlation between main members of the MAPK pathway and the CDKN1A-cell cycle regulators regulatory axis. Red corresponds to negative correlations, blue to positive correlations

To complete our comprehensive overview, we added the full MAPK pathway (Fig. 7). This pathway was not further explored because the correlation matrix (Fig. 8) suggested less impact of MAPK signaling on CDKN1A and the major cell cycle regulators, compared to the IGFBPs-AKT-FOXO1-CDKNA1 pathway. Nevertheless, the role of this pathway in FOXO inhibition and the significant correlation between NRAS and IGFBP7 make it worthwhile to further explore the regulatory effects of MAPK signaling on FOXO1 in GCs and to understand how IGFBP7 may regulate this pathway.

## Discussion

The sows subjected to feed restriction during lactation experienced a severe energy burden, which was confirmed by a higher percentage body weight loss and loin muscle depth loss compared to FF sows. An absence of backfat depth loss suggests that the mobilization of body reserves in these young sows primarily consisted of muscle proteins, and less of adipose tissue and may be explained by the lean phenotype of this breed of sows [5, 38]. The feed restriction also affected follicular development, assessed in the mid-follicular phase, as shown by a smaller ovary size and a smaller size of the largest healthy follicle. Having said this we cannot completely exclude that despite the use of a relatively large sample size (FF, *n* = 13; RES, *n* = 14) to minimize random variation and enhance the detection of true effects and relationships, and the standardized selection of the largest healthy follicle per ovary, the inherent variation in size of the follicles within the pool of 15 largest follicles may influence the differences in average ovary and follicle size between the FF and RES sows. While selecting multiple large healthy follicles might reduce this difference, it is unfortunately not feasible in practice. As multiple large follicles often reside on different focal planes, focusing on the precise sectioning of one follicle frequently necessitates the sacrifice or over-trimming of others. This is a technical limitation. We propose that these smaller follicles, characterized by a lower follicular fluid volume [5], are functionally compromised.

We previously showed that RES follicles have lower follicular fluid concentrations of key steroids (e.g., β-estradiol, progestins, and androgens) [5]. These analyses were performed on follicular fluid pooled from the 15 largest follicles per ovary, preventing direct correlations to the gene and protein expression changes made here. Beyond these steroidogenic differences, our previous results also show a reduced number of RES zygotes reaching the metaphase stage after in vitro maturation, and a higher incidence of polyspermy after in vitro fertilization [5], exemplifying the associated reduced reproductive performance of the RES sows.

We employed LCM to isolate pure mural GCs and used UMI-based RNA sequencing for accurate gene expression quantification, an approach that enables a comprehensive analysis of the differential transcriptomic responses of mural GCs between the nutritional energy-restricted and control groups. To identify molecular processes in GCs within the transcriptome responses, we performed functional enrichment analysis and WGCNA on our GC-specific RNA-seq data. This complementary approach reveals that cell cycle-related pathways are the most significant upregulated pathways in the RES GCs. Additionally, analysis of DEGs shows that proliferation markers *PCNA* and *MKI67* are highly expressed, while the expression of the differentiation marker *CYP19A1* is lower in RES GCs. These results, together with increased *INHBB* and decreased angiopoietin 1 (*ANGPT1*) expression, indicate that the RES GCs are in a more proliferative and less developed (differentiated) state, compared with the FF GCs. This agrees with the lower volume of follicular fluid and lower concentrations of steroid hormones, such as β-estradiol, in the follicular fluid of RES follicles [5]. The results of the present study align well with a previous comparison of the transcriptome profile of porcine GCs of smaller and larger healthy antral follicles within the same follicle pool. Despite the lower depth of these older data, the top-10 regulated pathways encompassed six cell cycle-related pathways, such as mitotic G2-G2/M phases, cell cycle, and M phase [19]. This agrees with the general understanding that small antral follicles are characterized by highly proliferating GCs. As antral follicular development continues, GCs are triggered to exit the cell cycle and initiate a program of terminal differentiation [39]. Promotion of the cell cycle typically inhibits differentiation, whereas cell differentiation is often accompanied by cell-cycle arrest [40], as we see in our data. Highly proliferative cells usually also upregulate glycolysis [36, 41] to provide biosynthesis intermediates to support growth and generate cell mass [42]. Consistent with this, a coordinated upregulation of nearly all glycolytic genes is observed in RES GCs. Interestingly, mitochondrial OXPHOS is concomitantly upregulated in RES GCs. In fact, most genes across all OXPHOS complexes were upregulated, together with genes involved in the TCA cycle, mitochondrial transcription, and mitochondrial translation (Fig. S6), implicating a broad upregulation of mitochondrial transcripts in RES GCs. This suggests an increase in mitochondrial metabolic capacity, likely to support the high in vivo energetic and biosynthetic demands. Recent evidence suggests that both OXPHOS and glycolysis are upregulated during the cell cycle to support DNA synthesis and chromosome segregation [42].

The current transcriptomic data, to some extent, reflect the combined effects of lactational energy stress and post-weaning ad libitum feeding on mural GC development, as the restricted sows still showed significantly lower IGF1 and creatinine levels [5]. However, the cross-sectional nature of the data, collected at only one time point, limits the ability to track dynamic developmental changes. We hypothesize that the growth and development of GCs in the RES group are delayed or halted compared to the FF group due to lactational energy insufficiency, with a compensatory catch-up response upon restoration of energy availability, as both groups were fed the same post-weaning diet. However, given that the timing of ovulation after weaning is fixed in sows [43], catch-up growth may not be sufficient to fully compensate, which could potentially contribute to reduced oocyte quality and impaired reproductive outcome [5].

To gain more insight into cell cycle regulation, we performed an in-depth analysis of the relevant genes involved in the cell cycle. The gene expression of major positive cell cycle regulators is significantly higher in RES GCs, whereas *CDKN1A* (P21), a cell cycle inhibitor, is significantly lower expressed in the RES GCs. CDKN1A is known to arrest cell cycle progression at the G1/S and G2/M transitions by inhibiting CDK4,6/cyclin-D and CDK2/cyclin-E, respectively, in response to a variety of stimuli [44]. It can also inhibit PCNA-dependent DNA replication through binding to PCNA [45]. The negative correlations between *CDKN1A* and key cell cycle regulators as well as with *PCNA* (Fig. S9), exclusively observed in the RES GCs, suggest that the downregulation of *CDKN1A* may be key in stimulating the proliferation of RES GCs and preventing them from exiting the cell cycle and initiating terminal differentiation.

Immunohistochemical analysis revealed that CDKN1A is exclusively localized in the cytoplasm rather than the nucleus of GCs. Furthermore, we observed that cytoplasmic CDKN1A levels are significantly higher in RES compared to FF GCs. At first glance, this may seem to contradict the hypothesis that high proliferation in RES GCs is driven by the reduced inhibitory effect of low CDKN1A expression. However, cytoplasmic CDKN1A has been suggested to facilitate cell proliferation [46], opposite to its role in the nucleus. Several studies have shown that CDKN1A exerts an anti-apoptotic role by binding to and inhibiting cleaved-caspase 3, as well as the apoptotic kinases apoptosis signal-regulating kinase 1 (ASK1) [47] and c-Jun N-terminal kinase (JNK) (also known as MAPK8) [48]. Importantly, Zhou et al. [49] reported that AKT phosphorylates CDKN1A and inhibits its antiproliferative effects by retaining it within the cytoplasm. Moreover, AKT-dependent phosphorylation of CDKN1A, retaining CDKN1A in the cytoplasm, is shown to promote endothelial cell proliferation [50]. In support of this, another study has demonstrated AKT to be an upstream negative regulator of CDKN1A-mediated cell cycle arrest [51]. Due to the limited amounts of material obtained from LCM, we are unable to determine whether the discrepancy between higher cytoplasmic protein levels and lower *CDKN1A* gene expression involves post-transcriptional regulation, or if these changes are related to changes in AKT activation. In line with this, we identified two miRNAs that potentially contribute to the post-transcriptional regulation of *CDKN1A* (unpublished data).

Besides AKT, the transcription factor FOXO1 can regulate *CDKN1A* expression by binding to its promoter [35, 52, 53]. This possibility is supported by the observed positive correlation between *FOXO1* and *CDKN1A* in RES sows. FOXO1 is a well-known downstream target of AKT [54, 55]. The insulin-like growth factor (IGF) receptor signaling pathway is one of the key upstream pathways regulating AKT activity [56]. This pathway can be stimulated by IGFs and insulin in response to growth signals and nutrients [57] and is negatively regulated by calorie restriction in many animal models [58]. We previously reported that serum as well as follicular fluid IGF1 levels are lower in the RES group compared to the FF group [5]. We now observe that the mRNA level of insulin receptor substrate 1 (IRS1) is significantly decreased under feed restriction conditions. The lower expression of *IRS1* aligns with a reduced activation of the IGF1-insulin signaling pathway and the higher expression of *FOXO1* and *CDKN1A*.

Several members of the insulin-like growth factor binding protein (IGFBP) system, including *IGFBP2*, *IGFBP4*, and *IGFBP7* show significantly lower expression in RES GCs. IGFBP2 and IGFBP4 can either stimulate or inhibit IGF-AKT signaling [59–61], while IGFBP7 negatively regulates IGF-AKT signaling [62, 63]. Correlation analysis shows that *IGFBP2*, *IGFBP4*, and *IGFBP7* are all strongly correlated with the identified major cell cycle regulators, with *IGFBP7* correlating with the majority of genes in the proposed regulatory axis. This is in line with the observation that IGFBP7, secreted into the follicular fluid, serves as an intraovarian factor that can negatively regulate GC differentiation [64]. Notably, the correlations of *IGFBP7* with the proposed regulatory axis are seen only in RES GCs. Recent studies have shown that ectopic overexpression of IGFBP7 can directly suppress phosphorylation-mediated activation of AKT at ser473 and thr308 [63, 65]. The lower expression of *IGFBP7* and its proposed direct effects on AKT may explain why AKT is higher expressed, while the insulin-IGF1 signaling pathway as a whole is lower expressed in RES GCs in the current pathway analyses. This, however, warrants further investigation taking AKT phosphorylation into account, since AKT function is primarily regulated by phosphorylation at various sites. These assumed direct effects of *IGFBP7* on AKT are in line with our observations that *AKT1* and *FOXO1* are not significantly correlated with each other and that *AKT1* is not correlated with *CDKN1A* in the RES GCs.

Of note, IGFBP7 has been detected in GCs [66–68] and differs from other IGFBPs in that it lacks conserved cysteines in the C domain [69], while it has a highly conserved follistatin sequence [70]. Follistatin can bind activin, which induces immature GCs to proliferate, and it promotes the expression of the FSH receptor and CYP19A1 [71]. While IGFBP7 can bind activin, it was shown to primarily bind and modulate IGF1 [72]. Human studies provide evidence that low levels of IGFBP7 are associated with energy restriction [73], aligning with our results. Based on these observations and our data, we propose that IGFBP7 might be the upstream sensor of whole-body energy changes, transducing signals through the (AKT)-FOXO1-CDKN1A-cell cycle axis to regulate GC proliferation. Regretfully, as the follicles were used for cryosection, it was not possible to obtain the corresponding follicular fluid of individual follicles, which is a limitation of this study. Nevertheless, strong correlations between follicle size and *IGFBP7* and other components of this regulatory axis support our hypothesis. Additional experiments are needed to determine whether IGFBP7 indeed serves as a central functional mediator linking energy metabolism and follicular development. A more in-depth analysis at the protein-level, as well as functional assays in GC models, may further substantiate our proposed IGFBPs-FOXO-CDKN1A-cell cycle regulatory axis.

## Conclusion

In this study, we utilized the physiological state of natural energy stress in lactating sows to explore how energy metabolism affects the molecular processes associated with a response of mural GCs to energy stress. Meanwhile, we assessed whether this energy insufficiency during lactation has a continuous impact following weaning and the return to normal feeding conditions. Phenotype results reveal that energy insufficiency during lactation delays or even halts follicular development and decreases oocyte competence. We propose a potential regulatory mechanism in which members of the IGFBP family, particularly IGFBP7, may function as potential sensors of energy status. Under conditions of energy insufficiency, their downregulation may modulate AKT and inhibit both FOXO1 and CDKN1A. This may promote sustained cell cycle progression, impacting their transition toward a differentiated state. Such an imbalance between proliferation and differentiation may ultimately compromise oocyte maturation and explains the reduced oocyte competence seen in the RES sows. While this is directly relevant for the pig, our results have a wider significance since the pig also serves as a human-relevant model for reproductive physiology.

## Supplementary Information

Additional file 1: Fig. S1 Schematic representation of the experimental design. Sows were full fed (FF, *n* = 13) or 50% feed restricted (RES, *n* = 14), during the last 2 weeks of lactation until weaning, and all were full fed for the two days thereafter. Pure mural granulosa cells (GCs) were collected for transcriptome analysis. Fig. S2 Follicle selection. Each sow’s right ovary was cut into two halves. To ensure (nearly all) large antral follicles were included, each half of the ovary was sectioned from three perspectives (top, middle and bottom), creating in a total of six sectioning planes. A cross was made on the left side to distinguish between the top and bottom halves. Next, each cutting plane was photographed before and after trimming against a grid paper as a scale reference using a Nikon D3300 camera. Follicle size was determined as the largest macroscopically visible diameter of the follicle using ImageJ software (v1.52). Images are from a random picked animal. Fig. S3 Follicle quality. The quality of the largest follicle, healthy or atretic, was evaluated using immunohistochemistry with an antibody against cleaved-caspase 3, a marker for apoptosis. The detailed protocol is included in Material and Method section. Representative immunofluorescence staining for a randomly picked animal is shown. Blue represents nuclear (DAPI) staining; Green represents cleaved-caspase 3 staining. Fig. S4 Validation of GC purity. Gene expression of established marker genes for theca cells (dark blue; *CYP11A1*, *CYP17A1*,*RET*, *COL14A1*, *PTCH2*, *DHCR24*, *BGN*), mural granulosa cells (orange; *CYB5A*, *INHA*, *INHBA*, *INHBB*,*GSTA1*, *FST*, *VCAN*), immune cells (sky blue; *CD68*, *CD16*,*CD19*), blood vessels (grey; *PDGFRB*, *CDH5*), and lymphatic vessels (brown; *LYVE1*). Fig. S5 The top GSEA GO terms ranked by normalized enrichment score (NES). The color of dots represents the adjusted *P*-value, and the size of dots represents the number of genes enriched. Fig. S6 The top GSEA KEGG pathways ranked by NES. The color of dots represents the adjusted *P*-value, and the size of dots represents the number of genes enriched. Fig. S7 Proliferation, differentiation and development markers. Violin plots of marker gene expression across individuals. Proliferation markers: PCNA and MKI67. Differentiation marker: CYP19A1. Mid-phase follicular development marker: INHBB. Late-phase follicular development marker: ANGPT1. Each dot represents an individual (*n* = 27). Fig. S8 Analysis of differential mitochondrial gene expression of RES versus FF pure mural granulosa cells (GCs). KEGG GSEA analysis of all mitochondrial genes according to Miticarta3.0 database. The top significantly enriched pathways ranked by GeneRatio. The color of dots represents the adjusted *P*-value, and the size of dots represents the number of genes enriched in respectively the pathway. Fig. S9 Correlation between CDKN1A (P21) and PCNA pure mural granulosa cells (GCs) gene expression level. Each dot represents an individual (*n* = 27). The *r* represents Pearson correlation coefficient. Table S1. RNA quality of the pure mural granulosa cells (GCs) largest follicles of each animal. Table S2. Statistics of clean reads and unique sequences in mRNA libraries.

Additional file 2: Table S3. Transcriptomic profiling of mitochondrial genes based on MitoCarta 3.0.

## Data Availability

The dataset(s) supporting the conclusions of this article is(are) available in the GEO (GSE298227) repository. All other data generated during the study are available from the corresponding author by request.

## Abbreviations

AKT1: RAC-alpha serine/threonine-protein kinase
BGN: Biglycan
CCNA1: Cyclin A1
CCNA2: Cyclin A2
CCNB1: Cyclin B1
CCNB2: Cyclin B2
CCNB3: Cyclin B3
CCNE2: Cyclin E2
CD19: CD19 molecule
CD16: Fc gamma receptor IIIa
CD68: Macrosialin
CDH5: Cadherin-5
CDK1: Cyclin-dependent kinase 1
CDK2: Cyclin-dependent kinase 2
CDK4: Cyclin-dependent kinase 4
CDKN1A: Cyclin-dependent kinase inhibitor 1A
COL14A1: Collagen type XIV alpha 1 chain
CYB5A: Cytochrome b5 type A
CYP11A1: Cytochrome P450 family 11 subfamily A member 1
CYP17A1: Cytochrome P450 family 17 subfamily A member 1
CYP19A1: Cytochrome P450 Family 19 subfamily A member 1
DEGs: Differentially expressed genes
DHCR24: Delta(24)-sterol reductase
FF: Full-fed
FOXO1: Forkhead box O1
FST: Follistatin
GCs: Granulosa cells
GO: Gene Ontology
GSEA: Gene set enrichment analysis
GSTA1: Glutathione S-transferase alpha 1
IGFBP2: Insulin-like growth factor binding protein 2
IGFBP4: Insulin-like growth factor binding protein 4
IGFBP7: Insulin-like growth factor binding protein 7
INHA: Inhibin alpha chain
INHBA: Inhibin beta A chain
INHBB: Inhibin beta B chain
KEGG: Kyoto Encyclopedia of Genes and Genomes
LCM: Laser capture microdissection
log_2_FC: Log_2_Foldchange
LYVE1: Lymphatic vessel endothelial hyaluronic acid receptor 1
MKI67: Proliferation marker protein Ki 67
NEB: Negative energy balance
NES: Normalized enrichment score
NRAS: GTPase NRas
OXPHOS: Oxidative phosphorylation
*P*_adj_: Adjusted *P*-value
PCA: Principal component analysis
PCNA: Proliferating cell nuclear antigen
PDGFRB: Platelet-derived growth factor receptor beta
PTCH2: Patched 2
RES: Restricted-fed
RET: Proto-oncogene tyrosine-protein kinase receptor
RIN: RNA Integrity Number
SD: Standard deviation
TBS: Tris-buffered saline
UMI: Unique molecular identifiers
VCAN: Versican core protein
WGCNA: Weighted correlation network analysis
