# Supplementary material for: Feed restriction affects follicular development by regulating cell cycle progression in porcine mural granulosa cells
Source: J Anim Sci Biotechnol. 2026 Jul 21;17:149. doi: 10.1186/s40104-026-01464-1 (PMC13386861; doi:10.1186/s40104-026-01464-1)
Supplement: Supplementary file 1 — Additional file 1: Fig. S1 Schematic representation of the experimental design. Sows were full fed (FF, n = 13) or 50% feed restricted (RES, n = 14), during the last 2 weeks of lactation until weaning, and all were full fed for the two days thereafter. Pure mural granulosa cells (GCs) were collected for transcriptome analysis. Fig. S2 Follicle selection. Each sow’s right ovary was cut into two halves. To ensure (nearly all) large antral follicles were included, each half of the ovary was sectioned from three perspectives (top, middle and bottom), creating in a total of six sectioning planes. A cross was made on the left side to distinguish between the top and bottom halves. Next, each cutting plane was photographed before and after trimming against a grid paper as a scale reference using a Nikon D3300 camera. Follicle size was determined as the largest macroscopically visible diameter of the follicle using ImageJ software (v1.52). Images are from a random picked animal. Fig. S3 Follicle quality. The quality of the largest follicle, healthy or atretic, was evaluated using immunohistochemistry with an antibody against cleaved-caspase 3, a marker for apoptosis. The detailed protocol is included in Material and Method section. Representative immunofluorescence staining for a randomly picked animal is shown. Blue represents nuclear (DAPI) staining; Green represents cleaved-caspase 3 staining. Fig. S4 Validation of GC purity. Gene expression of established marker genes for theca cells (dark blue; CYP11A1, CYP17A1,RET, COL14A1, PTCH2, DHCR24, BGN), mural granulosa cells (orange; CYB5A, INHA, INHBA, INHBB,GSTA1, FST, VCAN), immune cells (sky blue; CD68, CD16,CD19), blood vessels (grey; PDGFRB, CDH5), and lymphatic vessels (brown; LYVE1). Fig. S5 The top GSEA GO terms ranked by normalized enrichment score (NES). The color of dots represents the adjusted P-value, and the size of dots represents the number of genes enriched. Fig. S6 The top GSEA KEGG pathways ranked by NES. Th [file 40104_2026_1464_MOESM1_ESM.docx]

**Feed restriction affects follicular development by regulating cell cycle progression in porcine mural granulosa cells**

Qi Yu, Anna F. Bekebrede, Natasja N.G. Costermans, Nicoline M. Soede, Katja J. Teerds, and Jaap Keijer

Journal of Animal Science and Biotechnology


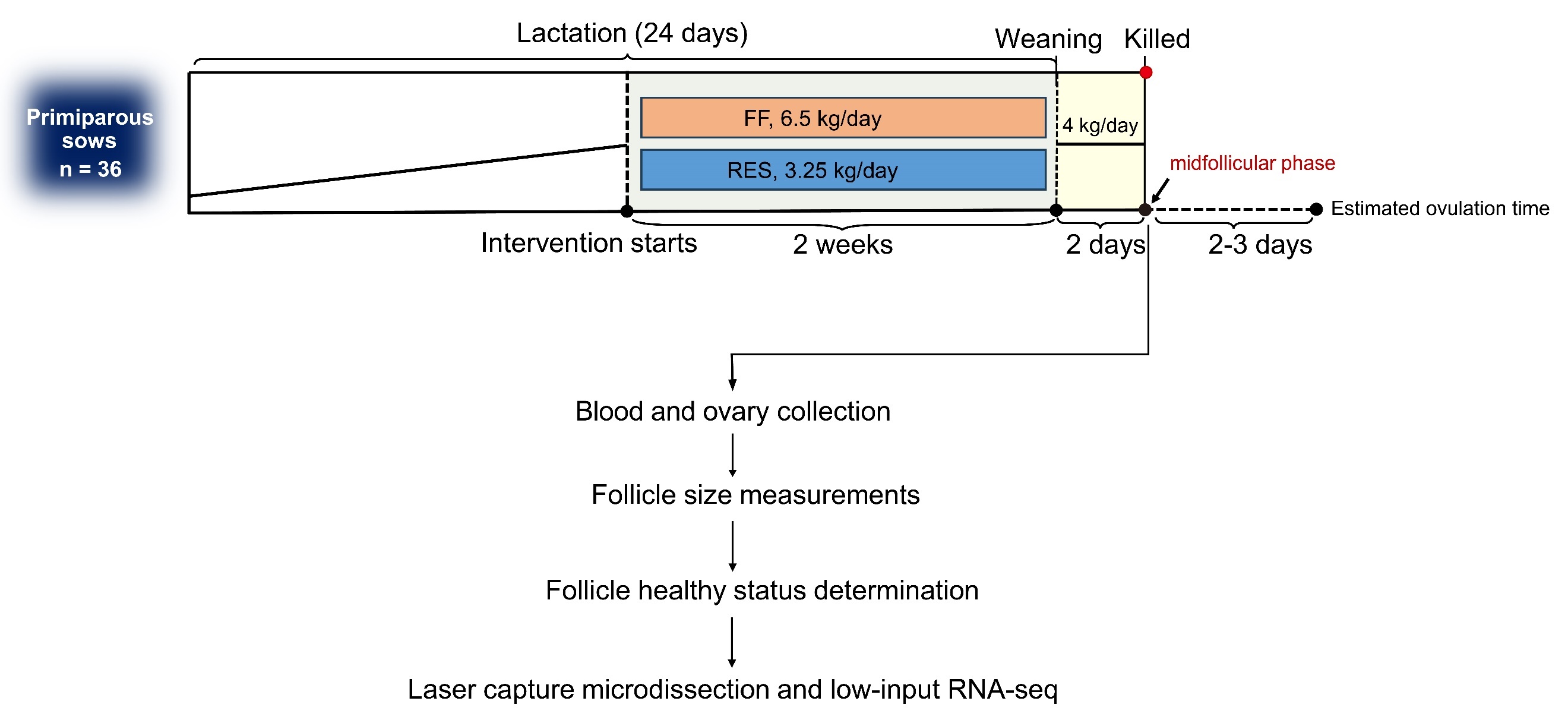


Fig. S1. **Schematic representation of the experimental design.** Sows were full fed (FF, n=14) or 50% feed restricted (RES, n=14), during the last 14 days of lactation until weaning, and all were full fed for the two days thereafter. Pure mural granulosa cells (GCs) were collected for transcriptome analysis.


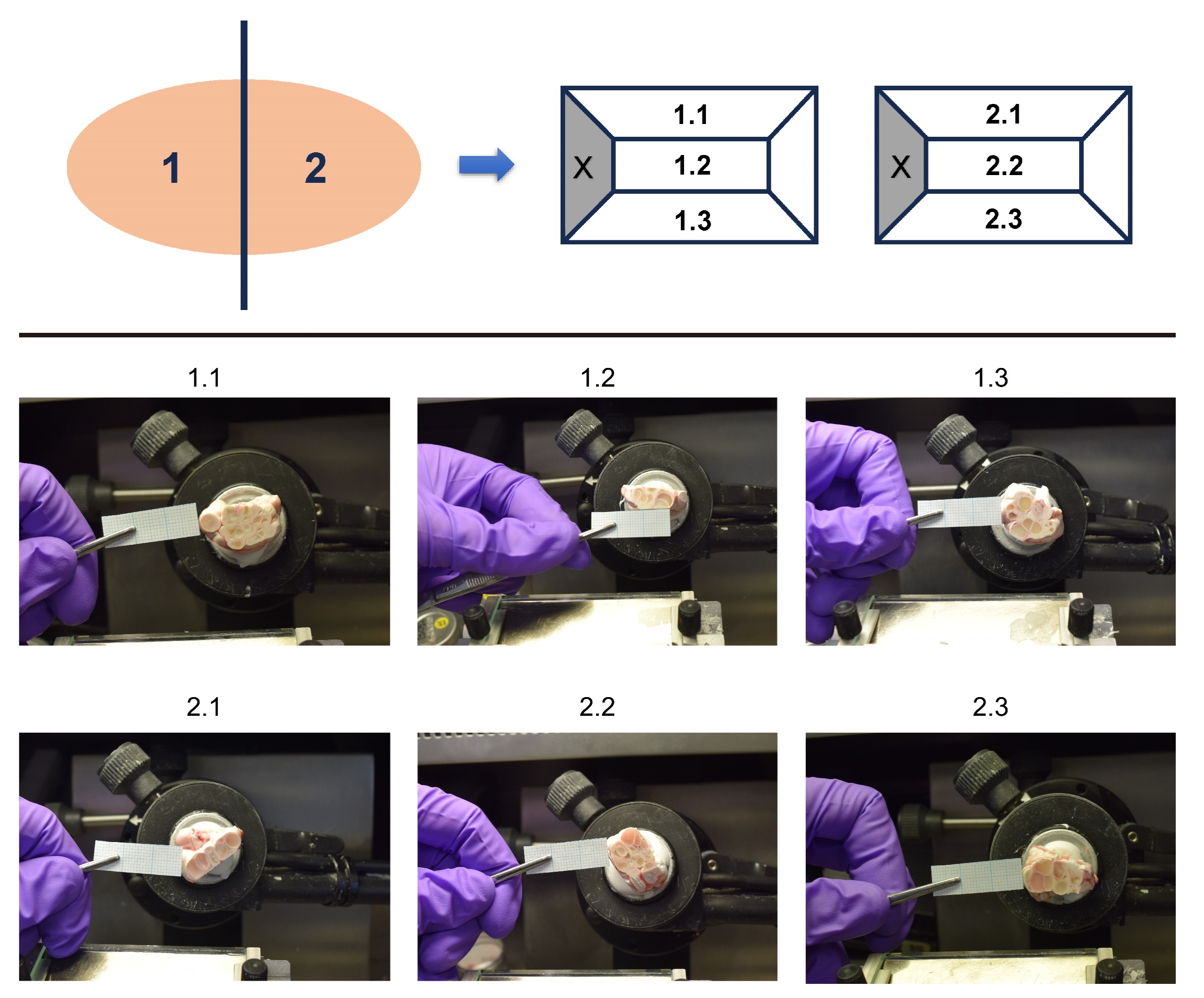


Fig. S2. **Follicle selection.** Each sow’s right ovary was cut into two halves. To ensure (nearly all) large antral follicles were included, each half of the ovary was sectioned from three perspectives (top, middle and bottom), creating in a total of six sectioning planes. A cross was made on the left side to distinguish between the top and bottom halves. Next, each cutting plane was photographed before and after trimming against a grid paper as a scale reference using a Nikon D3300 camera. Follicle size was determined as the largest macroscopically visible diameter of the follicle using ImageJ software (v1.52). Images are from a random picked animal.

**
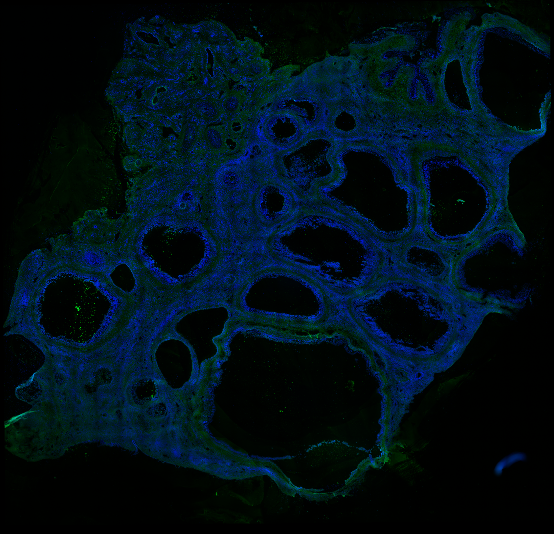

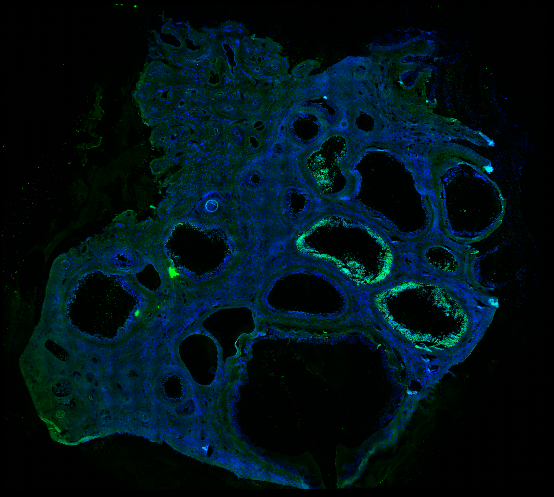
**

Atretic follicle

Atretic follicle

Target follicle

IgG (negative control) (negative control)

Cleaved-caspase-3 (apoptosis marker)

Fig. S3. **Follicle quality.** The quality of the largest follicle, healthy or atretic, was evaluated using immunohistochemistry with an antibody against cleaved Caspase 3, a marker for apoptosis. The detailed protocol is included in Material and Method section. Representative immunofluorescence staining for a randomly picked animal is shown. Blue represents nuclear (DAPI) staining; Green represents cleaved-caspase 3 staining.


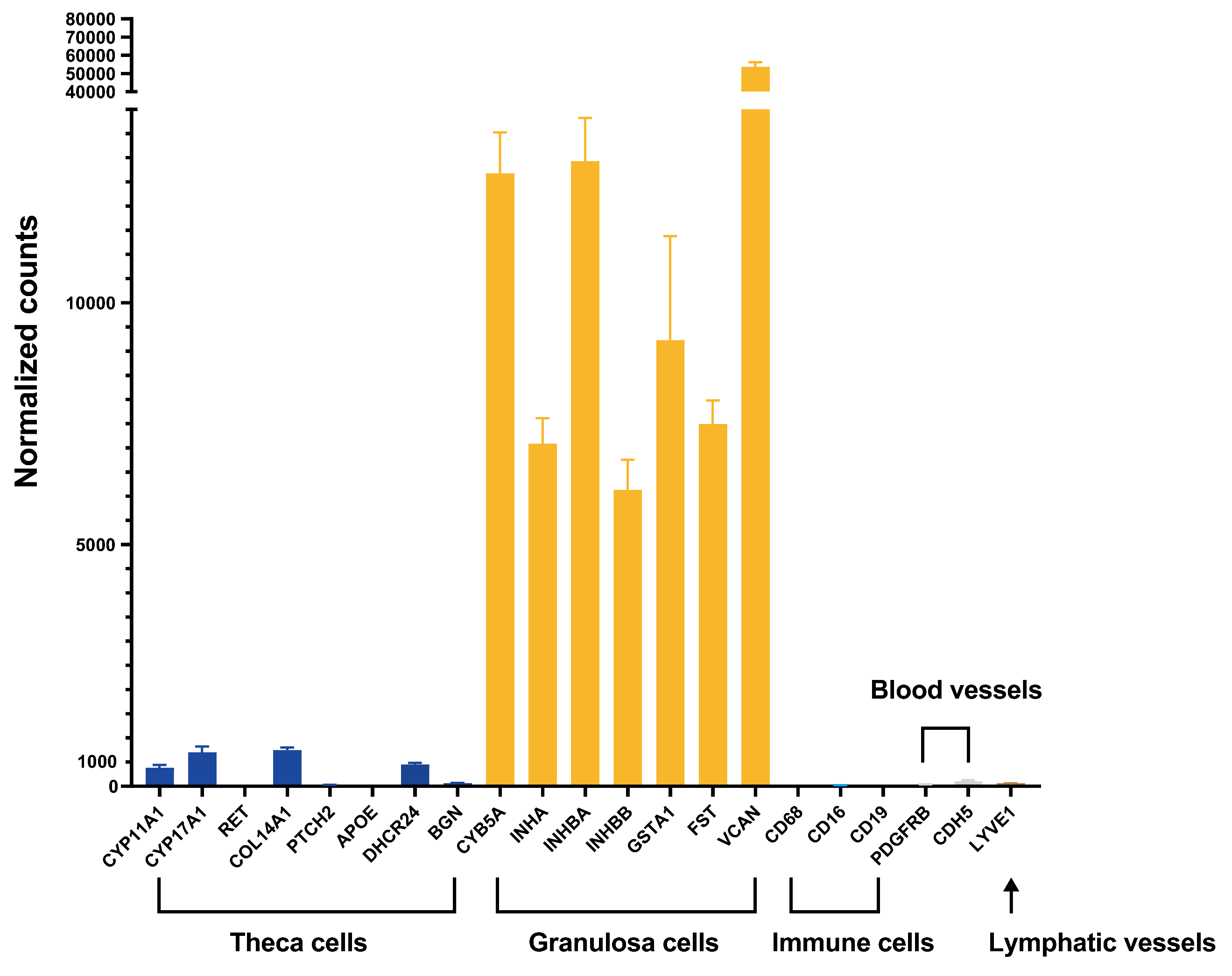


Fig. S4. **Validation of GC purity**. Gene expression of established marker genes for theca cells (dark blue; *CYP11A1*, *CYP17A1*, *RET*, *COL14A1*, *PTCH2*, *DHCR24*, *BGN*), mural granulosa cells (orange; *CYB5A*, *INHA*, *INHBA*, *INHBB*, *GSTA1*, *FST*, *VCAN*), immune cells (sky blue; *CD68*, *CD16*, *CD19*), blood vessels (grey; *PDGFRB*, *CDH5*), and lymphatic vessels (brown; *LYVE1*).


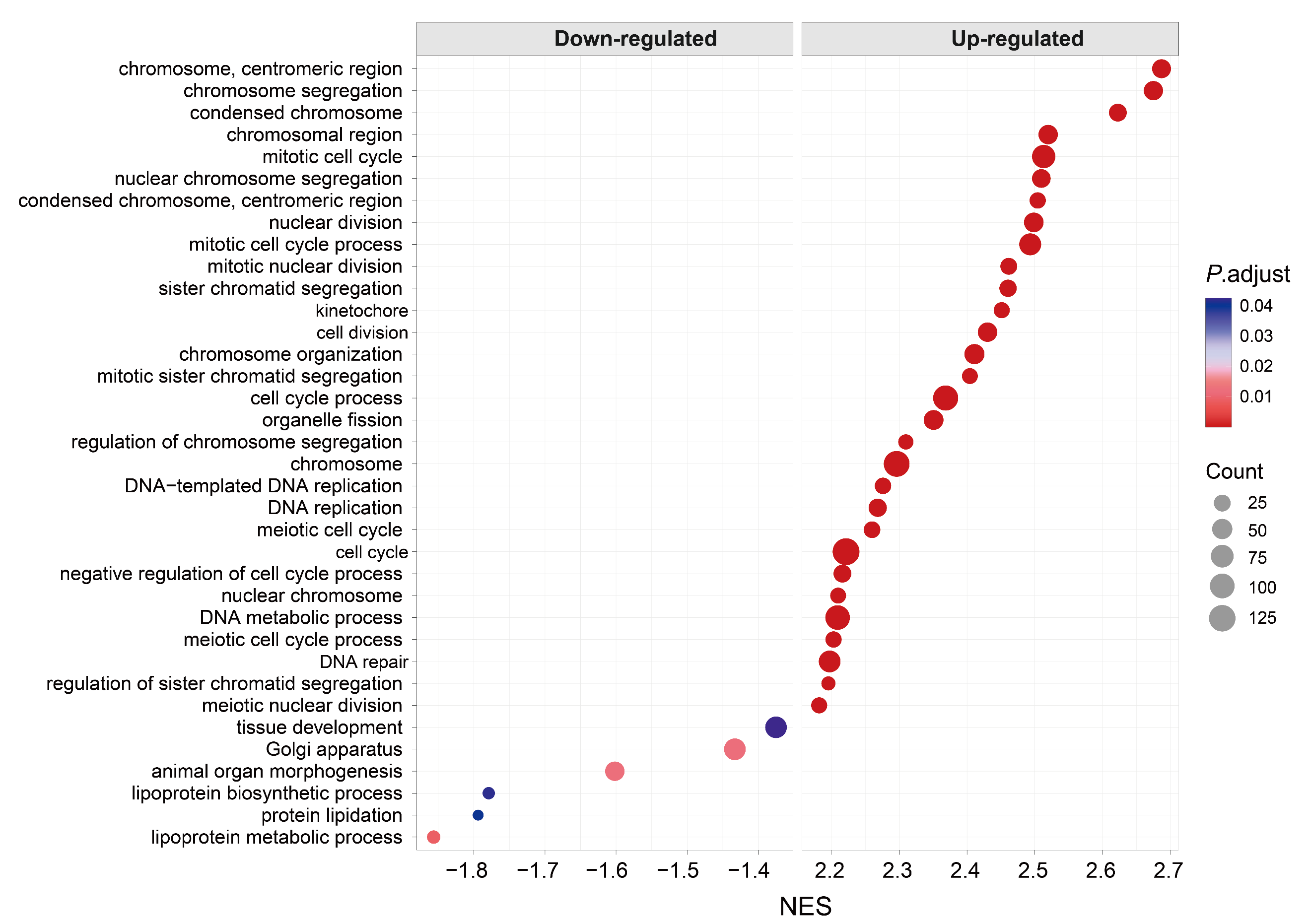


Fig. S5. **The top GSEA GO terms ranked by normalized enrichment score (NES).** The color of dots represents the adjusted *P*-value, and the size of dots represents the number of genes enriched.


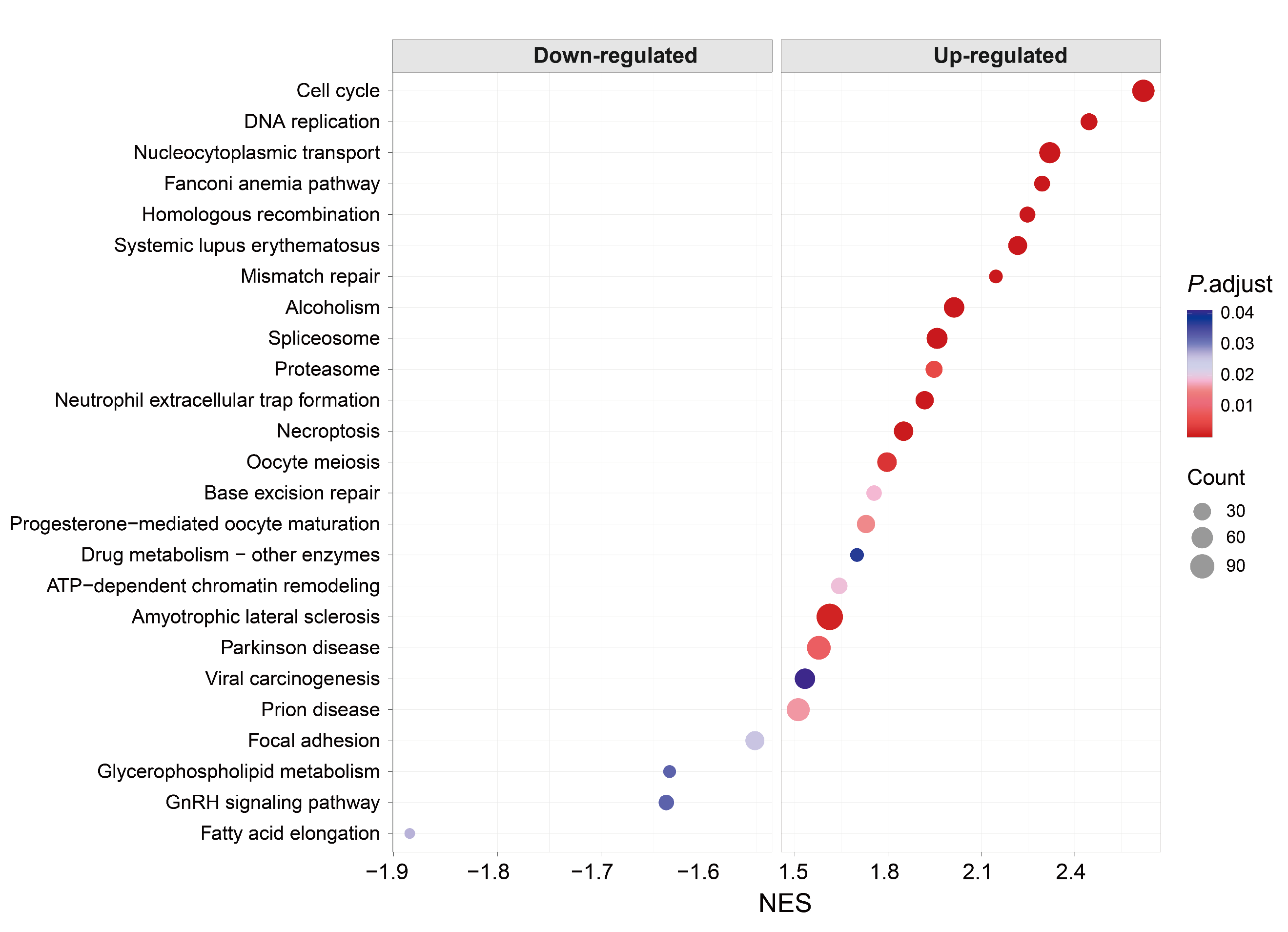


Fig. S6. **The top GSEA KEGG pathways ranked by NES**. The color of dots represents the adjusted *P*-value, and the size of dots represents the number of genes enriched.


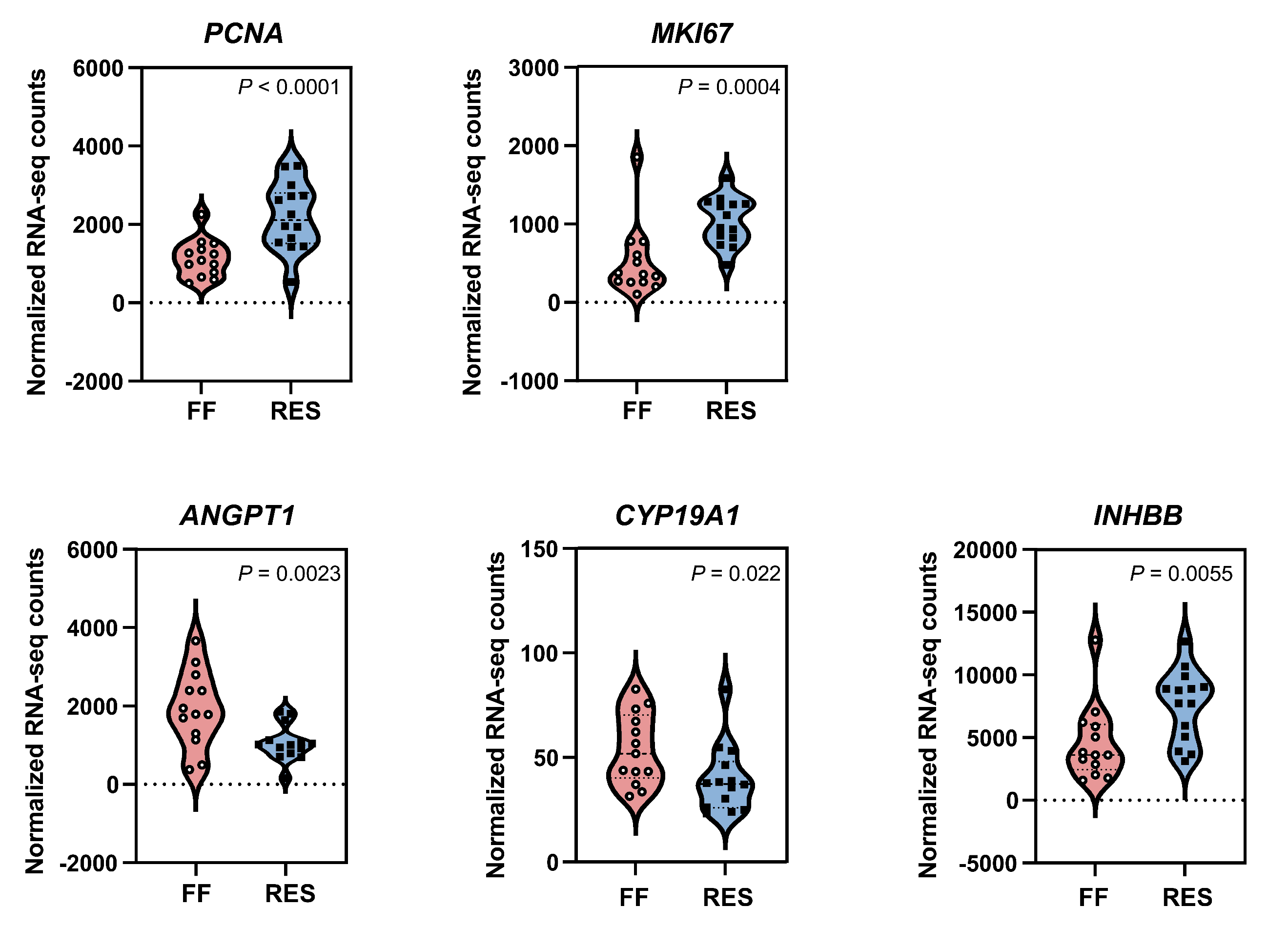


Fig. S7. **Proliferation, differentiation and development markers.** Violin plots of marker gene expression across individuals. Proliferation markers: *PCNA* and *MKI67*. Differentiation marker: *CYP19A1*. Mid-phase follicular development marker: *INHBB*. Late-phase follicular development marker: *ANGPT1*. Each dot represents an individual (n = 27).


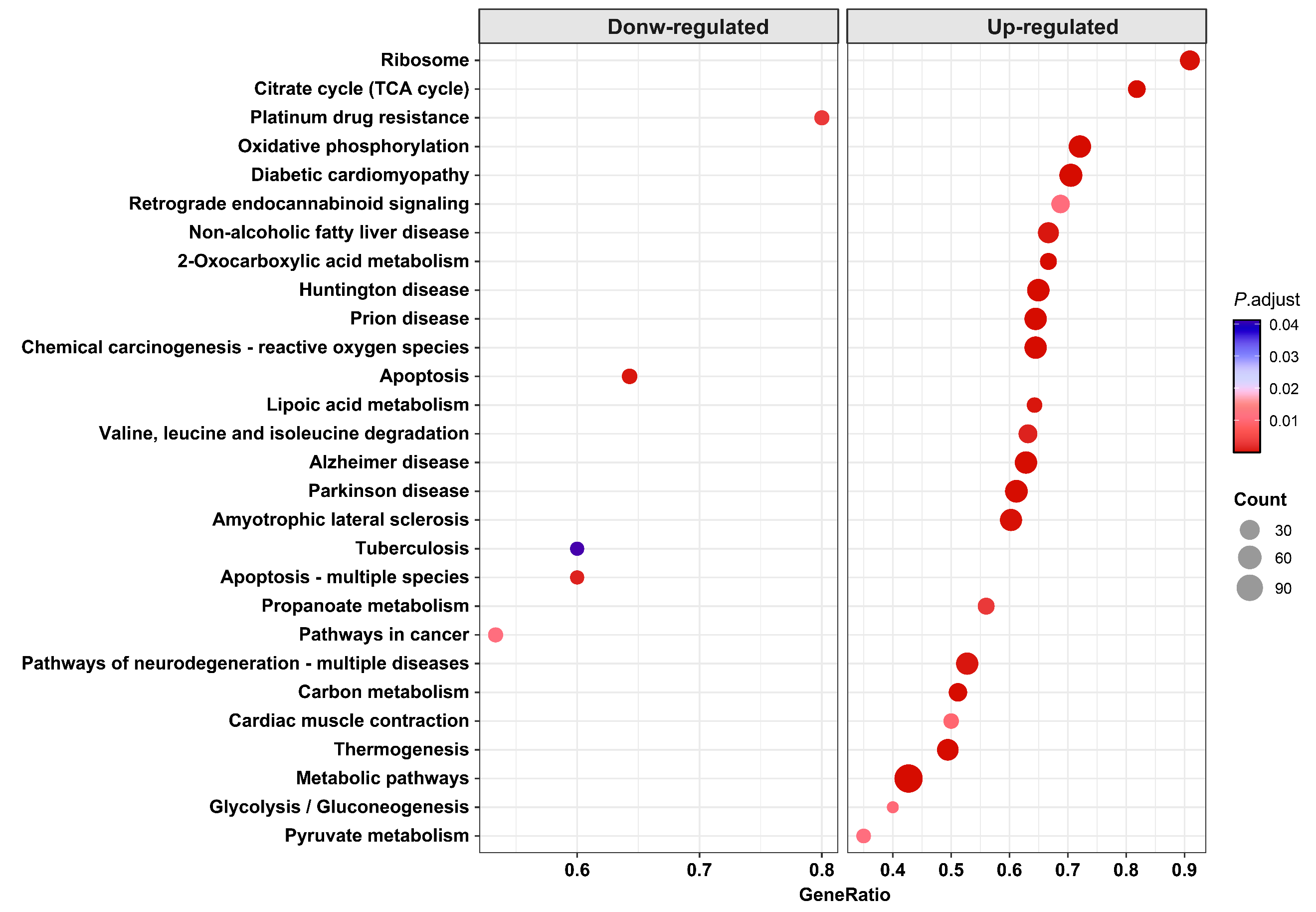


Fig. S8. **Analysis of differential mitochondrial gene expression of RES versus FF pure mural granulosa cells (GCs).** KEGG GSEA analysis of all mitochondrial genes according to Miticarta3.0 database. The top significantly enriched pathways ranked by GeneRatio. The color of dots represents the adjusted p value, and the size of dots represents the number of genes enriched in respectively the pathway.


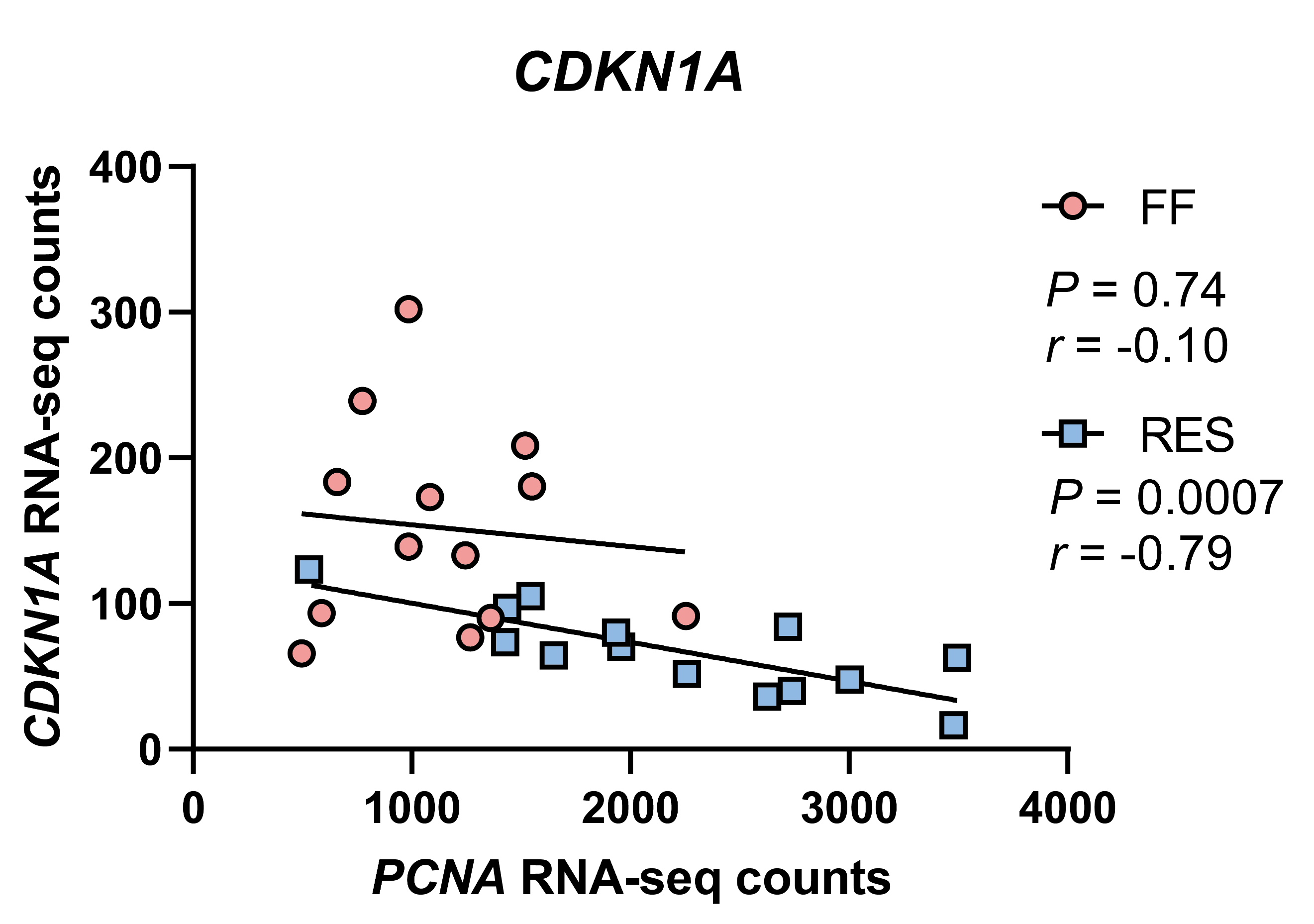


Fig. S9. **Correlation between *CDKN1A* (P21) and *PCNA* pure mural granulosa cells (GCs) gene expression level**. Each dot represents an individual (n = 27). The r represents Pearson correlation coefficient.

**Table S1**

RNA quality of the pure mural granulosa cells (GCs) largest follicles of each animal

| **Animal**  **(FF^a^)** | **Concentration**  **(pg/µl)** | **RIN value** | **28S/18S** | **Animal**  **(RES^b^)** | **Concentration**  **(pg/µl)** | **RIN value^c^** | **28S/18S** |
| --- | --- | --- | --- | --- | --- | --- | --- |
| FF-1 | 355 | 7.7 | 0.7 | RES-1 | 787 | 7.8 | 1.0 |
| FF-2 | 627 | 7.7 | 1.0 | RES-2 | 1273 | 7.0 | 0.7 |
| FF-3 | 578 | 7.4 | 0.8 | RES-3 | 5354 | 6.3 | 0.9 |
| FF-4 | 1329 | 7.3 | 1.1 | RES-4 | 739 | 7.0 | 1.2 |
| FF-5 | 1304 | 7.0 | 0.8 | RES-5 | 2070 | 6.8 | 0.8 |
| FF-6 | 1438 | 7.4 | 0.9 | RES-6 | 644 | 8.0 | 0.8 |
| FF-7 | 1742 | 7.2 | 0.9 | RES-7 | 440 | 7.7 | 0.7 |
| FF-8 | 1268 | 7.0 | 1.0 | RES-8 | 825 | 7.2 | 0.7 |
| FF-9 | 468 | 7.1 | 1.1 | RES-9 | 532 | 8.2 | 1.0 |
| FF-10 | 1830 | 5.5 | 0.2 | RES-10 | 1271 | 6.6 | 0.6 |
| FF-11 | 309 | 7.9 | 1.2 | RES-11 | 433 | 8.3 | 1.1 |
| FF-12 | 566 | 7.2 | 0.8 | RES-12 | 429 | 8.1 | 1.0 |
| FF-13 | 1228 | 7.1 | 1.0 | RES-13 | 667 | 7.4 | 0.8 |
| FF-14 | 1244 | 7.1 | 0.8 | RES-14 | 979 | 7.0 | 0.7 |

^a^ Full-fed group

^b^ Restricted-fed group

^c^ RIN: RNA Integrity Number

**Table S2**

Statistics of clean reads and unique sequences in mRNA libraries

| **Sample name** | **Clean Reads** | **Total Mapped** | **Multiple Mapped** | **Uniquely Mapped** | **GC, %** | **Q20, %** | **Q30, %** |
| --- | --- | --- | --- | --- | --- | --- | --- |
| FF^a^-1 | 36,669,381 | 32,192,616 (87.79%) | 3,381,338 (9.22%) | 28,811,278 (78.57%) | 42.25 | 97.92 | 94.16 |
| FF-2 | 36,691,903 | 29,889,540 (87.94%) | 2,378,004 (6.48%) | 29,889,540 (81.46%) | 41.04 | 98.29 | 94.84 |
| FF-3 | 33,578,328 | 26,837,151 (87.27%) | 2,467,645 (7.35%) | 26,837,151 (79.92%) | 40.89 | 98.36 | 95.07 |
| FF-4 | 35,771,378 | 31,645,133 (88.46%) | 2,530,134 (7.07%) | 29,114,999 (81.39%) | 41.5 | 98.19 | 94.53 |
| FF-5 | 36,026,081 | 29,174,924 (88.01%) | 2,530,770 (7.02%) | 29,174,924 (80.98%) | 41.7 | 97.81 | 93.84 |
| FF-6 | 38,882,649 | 34,204,573 (87.97%) | 3,303,591 (8.50%) | 30,900,982 (79.47%) | 41.31 | 98.28 | 94.83 |
| FF-7 | 35,646,389 | 31,762,098 (89.10%) | 1,655,389 (4.64%) | 30,106,709 (84.46%) | 40.38 | 98.25 | 94.7 |
| FF-8 | 40,129,888 | 35,253,805 (87.85%) | 2,553,927 (6.36%) | 32,699,878 (81.49%) | 41.07 | 97.79 | 93.78 |
| FF-9 | 37,689,830 | 32,661,179 (86.66%) | 2,596,003 (6.89%) | 30,065,176 (79.77%) | 40.91 | 98.33 | 95.01 |
| FF-10 | 39,676,588 | 34,571,399 (87.13%) | 4,710,010 (11.87%) | 29,861,389 (75.26%) | 42.61 | 97.81 | 93.88 |
| FF-11 | 37,514,005 | 32,988,773 (87.94%) | 2,797,119 (7.46%) | 30,191,654 (80.48%) | 41.7 | 98.24 | 94.7 |
| FF-12 | 37,349,364 | 32,457,354 (86.90%) | 2,433,974 (6.52%) | 30,023,380 (80.39%) | 40.58 | 97.87 | 94.03 |
| FF-13 | 40,012,910 | 35,095,271 (87.71%) | 2,905,036 (7.26%) | 32,190,235 (80.45%) | 41.27 | 97.84 | 93.93 |
| FF-14 | 34,820,793 | 30,455,926 (87.46%) | 1,890,238 (5.43%) | 28,565,688 (82.04%) | 40.69 | 98.32 | 94.89 |
| RES^b^-1 | 37,896,875 | 33,228,362 (87.68%) | 2,568,129 (6.78%) | 30,660,233 (80.90%) | 41.68 | 98.32 | 94.93 |
| RES-2 | 39,268,935 | 34,417,436 (87.65%) | 2,603,227 (6.63%) | 31,814,209 (81.02%) | 40.62 | 97.89 | 94.05 |
| RES-3 | 39,564,661 | 35,531,414 (88.57%) | 4,258,666 (10.62%) | 31,272,748 (77.96%) | 42.55 | 97.17 | 92.66 |
| RES-4 | 39,697,610 | 34,911,596 (87.94%) | 2,606,441 (6.57%) | 32,305,155 (81.38%) | 41.68 | 98.28 | 94.79 |
| RES-5 | 40,115,725 | 34,945,893 (88.33%) | 3,386,340 (8.56%) | 31,559,553 (79.77%) | 43.62 | 97.25 | 92.9 |
| RES-6 | 37,825,149 | 33,101,495 (87.51) | 2,723,633 (7.20%) | 30,377,862 (80.31%) | 41.11 | 98.31 | 94.88 |
| RES-7 | 40,758,535 | 35,327,204 (86.67%) | 2,558,658 (6.28%) | 32,768,546 (80.40%) | 40.91 | 97.93 | 94.18 |
| RES-8 | 40,783,202 | 35,706,390 (87.55%) | 2,679,283 (6.57%) | 33,027,107 (80.98%) | 40.93 | 97.87 | 93.97 |
| RES-9 | 35,768,247 | 31,210,228 (87.26%) | 2,656,933 (7.43%) | 28,553,295 (79.83%) | 41.56 | 98.3 | 94.84 |
| RES-10 | 33,420,856 | 29,346,224 (87.81%) | 2,234,394 (6.69%) | 27,111,830 (81.12%) | 41.69 | 98.37 | 95.08 |
| RES-11 | 40,790,870 | 35,840,197 (87.86%) | 2,545,584 (6.24%) | 33,294,613 (81.62%) | 41.56 | 98.32 | 94.89 |
| RES-12 | 33,595,222 | 29,490,792 (87.78%) | 2,208,740 (6.57%) | 27,282,052 (81.21%) | 41.72 | 98.23 | 94.66 |
| RES-13 | 39,245,421 | 34,333,939 (87.49%) | 3,497,828 (8.91%) | 30,836,111 (78.57%) | 41.39 | 97.81 | 93.83 |
| RES-14 | 39,280,481 | 34,125,706 (86.88%) | 2,797,519 (7.12%) | 31,328,187 (79.76%) | 40.99 | 97.94 | 94.22 |

^a^ Full-fed group

^b^ Restricted-fed group
